# Macrocycle peptides delineate locked-open inhibition mechanism for microorganism phosphoglycerate mutases

**DOI:** 10.1038/ncomms14932

**Published:** 2017-04-03

**Authors:** Hao Yu, Patricia Dranchak, Zhiru Li, Ryan MacArthur, Matthew S. Munson, Nurjahan Mehzabeen, Nathan J. Baird, Kevin P. Battalie, David Ross, Scott Lovell, Clotilde K. S. Carlow, Hiroaki Suga, James Inglese

**Affiliations:** 1 Department of Chemistry, Graduate School of Sciences, The University of Tokyo, Tokyo 113-0033, Japan; 2 National Center for Advancing Translational Sciences, National Institutes of Health, Rockville, Maryland 20850, USA; 3 Division of Genome Biology, New England Biolabs, Ipswich, Massachusetts 01938, USA; 4 National Institute of Standards and Technology, Gaithersburg, Maryland 20899, USA; 5 Proton Structure Laboratory, Structural Biology Center, University of Kansas, Lawrence, Kansas 66047, USA; 6 National Heart, Lung and Blood Institute, National Institutes of Health, Bethesda, Maryland 20892, USA; 7 IMCA-CAT Advanced Photon Source, Argonne National Laboratory, Argonne, Illinois 60439, USA

## Abstract

Glycolytic interconversion of phosphoglycerate isomers is catalysed in numerous pathogenic microorganisms by a cofactor-independent mutase (iPGM) structurally distinct from the mammalian cofactor-dependent (dPGM) isozyme. The iPGM active site dynamically assembles through substrate-triggered movement of phosphatase and transferase domains creating a solvent inaccessible cavity. Here we identify alternate ligand binding regions using nematode iPGM to select and enrich lariat-like ligands from an mRNA-display macrocyclic peptide library containing >10^12^ members. Functional analysis of the ligands, named ipglycermides, demonstrates sub-nanomolar inhibition of iPGM with complete selectivity over dPGM. The crystal structure of an iPGM macrocyclic peptide complex illuminated an allosteric, locked-open inhibition mechanism placing the cyclic peptide at the bi-domain interface. This binding mode aligns the pendant lariat cysteine thiolate for coordination with the iPGM transition metal ion cluster. The extended charged, hydrophilic binding surface interaction rationalizes the persistent challenges these enzymes have presented to small-molecule screening efforts highlighting the important roles of macrocyclic peptides in expanding chemical diversity for ligand discovery.

## Abstract (web summary)

River blindness, a disease affecting millions throughout the tropics, is caused by parasitic worms. Here, Yu *et al*. report the discovery and structural characterization of potent macrocyclic peptide inhibitors of iPGM, a nematode-specific phosphoglycerate mutase, as potential leads for novel antimicrobial agents.

Nematode worms are the most abundant animal on earth^1^ and are found in widely different environments. They can be free-living or parasitic, infecting plants, animals and humans. Parasitic nematode infection in humans can lead to a number of devastating diseases. Lymphatic filariasis and onchocerciasis are neglected tropical diseases caused by filarial nematode parasites that are transmitted to humans by insects. Collectively, they afflict 150 million people in over 80 countries and threaten the health of over 1.5 billion^2^. These infections are responsible for extreme infirmity, social stigma and severe economic consequences. The lymphatic dwelling parasites such as *Wuchereria bancrofti* and *Brugia malayi* are the cause of lymphedema, hydrocele and in the most extreme cases, elephantiasis. Infection with *Onchocerca volvulus* results in severe dermatitis and blindness. The mainstay of filarial disease control for several years has been a limited number of drugs, predominantly ivermectin together with albendazole (where onchocerciasis is endemic) or diethylcarbamazine citrate (where onchocerciasis is not present). These compounds mainly target the larval stages and require annual or semi-annual administration. Furthermore, there are reports of drug resistance emerging^3^,^4^.

Enzymes essential for nematode survival but absent from humans represent potential targets for intervention. Essential nematode genes have been identified using comparative genomic studies of the free-living nematode *Caenorhabditis elegans* based on an algorithm designed to evaluate criteria such as *Homo sapiens* homology and life stage expression profile. As a result several novel drug targets in filarial parasites have been proposed. Among the highest ranking is cofactor-independent phosphoglycerate mutase (iPGM) (EC 5.4.2.1)^5^. Silencing of *ipgm* in *C. elegans*^6^ and *B. malayi*^7^ leads to nematode death, demonstrating the importance of this enzyme in nematode viability and, therefore, its potential as an anthelmintic drug target.

Phosphoglycerate mutases (PGMs) catalyse the interconversion of 2- and 3-phosphoglycerate (2-PG; 3-PG) in the glycolytic and gluconeogenic pathways. Although these pathways are highly conserved among different organisms, two distinct PGM isoenzymes are known to exist, namely iPGM and cofactor-dependent phosphoglycerate mutase (dPGM). The enzymes have no amino-acid sequence similarity and differ in their mechanism of catalysis (Fig. 1a). iPGM is comprised of ∼510 amino acids and facilitates the intramolecular transfer of the phosphoryl group on the monophosphoglycerates through a phosphoserine intermediate and is the sole PGM in nematode^8^,^9^. In contrast, dPGM, the isozyme found in humans is comprised of ∼250 amino acids, and catalyses the intermolecular transfer of the phosphoryl group between the monophosphoglycerates and the cofactor (2,3-diphosphoglycerate) through a phosphorylhistidine intermediate^10^. While the two forms of PGM are distinct isozymes, the amino-acid sequence of each isozyme family is conserved, when present, from bacteria to higher eukaryotes^11^. The completely distinct structures and catalytic mechanism of iPGM and dPGM enzymes offer great promise for the discovery of inhibitors with high selectivity for the nematode enzymes. Furthermore, the high similarity in primary sequence and catalytic properties among the iPGMs suggests that a single inhibitor could be effective against a range of parasitic and microbial enzymes.

Here we report a series of cyclic peptides and analogues that exhibit potent and isozyme-selective inhibition against iPGM orthologues. The parental peptide, we named ipglycermide, was discovered from a library containing over a trillion cyclic peptide members, each displayed on a cognate mRNA template. Ipglycermide has a unique lariat structure, where the ring peptide consists of eight amino-acid residues, one of which is D-tyrosine, closed by a thioether bond and a seven residue pendant extension which places L-cysteine (Cys) at the C terminus. Our 1.95 Å co-crystal structure reveals allosteric binding to a heretofore unknown site rich in charged side chains but devoid of a large hydrophobic surface. Ipglycermide binding constrains the dynamic movement between the phosphatase and transferase protein domains required for catalysis.

Our study suggests that the chemical diversity afforded by nucleic acid encoded cyclic peptides effectively maps putative, or even previously unknown, protein surfaces for drug discovery where small-molecule screening probes have failed^12^. In addition, we show that macrocyclic peptides capture dynamic protein domains to allosterically impose constraints on functional motions. Overall, ipglycermide inhibition of iPGM will serve as an effective model for discovery and inhibition of the many challenging targets lacking deep hydrophobic binding pockets, and instead principally characterized by conformationally flexible hydrophilic surfaces^13^.

## Results

### RaPID display affinity selection

A recently reported attempt to obtain small-molecule inhibitors against iPGM from a combined library of 380,000 compounds by Genzyme Corporation and the National Center for Drug Screening in Shanghai resulted in only two low-potency compounds, apparent metal ion chelators^12^. Given the evident refractory nature of iPGM toward small-molecule inhibition outside of metal ion ligands, and the difficulty of identifying chemotypes of the alkaline phosphatase superfamily enzyme class from high-throughput screening (HTS) with sub-micromolar optimization potential^14^, we approached inhibitor identification through the complementary method of affinity selection using an *in vitro* display system, referred to as RaPID (random nonstandard peptides integrated discovery).

**Figure 1:**
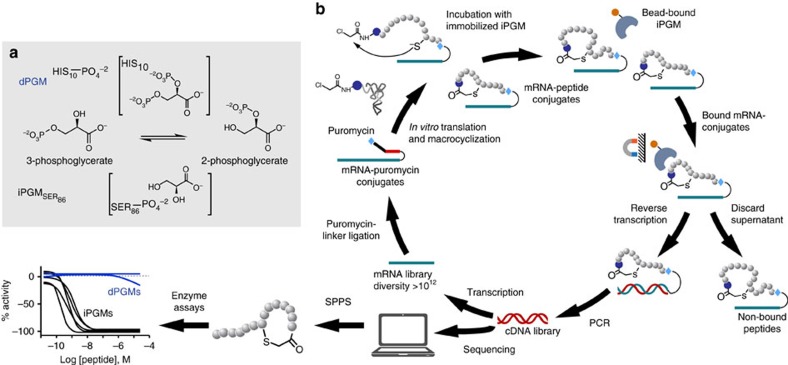
Species-dependent phosphoglycerate mutase catalytic mechanisms and overview of affinity selection. (**a**) Isomerization catalysed by PGMs illustrating the phosphohistidine enzyme/2,3-phosphoglycerate intermediate of human cofactor-dependent PGM (top) and the phosphoserine enzyme intermediate of *C. elegans* cofactor-independent PGM. (**b**) Random nonstandard peptide integrated discovery (RaPID) begins with an mRNA library encoding trillions of potential peptides 6–14 amino acids in length. The mRNA library is ligated to an adapter incorporating the amino nucleoside, puromycin. The flexible *in vitro* translation (FIT) system is used to create the peptide library with an L- or D-*N*-chloroacetyl tyrosine (dark blue sphere) charged initiator tRNA and 19 proteogenic amino acids (grey spheres), methionine is excluded as its tRNA is charged with the chloroacetyl tyrosine. Incorporation of a cysteine during translation results in macrocyclization via thiolate nucleophilic attack on the chloroacetyl electrophile. After incubating with the library the beads are washed to enrich the bound conjugates which are then reverse transcribed and amplified via PCR. PCR products are transcribed to mRNAs and the process is repeated or PCR products sequenced to reveal the peptide sequences captured. Peptides from these sequences are then produced in milligram quantity using solid-phase peptide synthesis (SPPS).

The RaPID system (Fig. 1b) enabled us to exploit the diverse molecular topology of macrocyclic peptide populations numbering in a trillion unique members and enrich for and amplify low abundance, high-affinity ligands^15^. We utilized a thioether-cyclic peptide library initiated with both L- or D-*N*-chloroacetyl tyrosine and performed ligand selection against iPGM protein from two nematode species, *B. malayi* and *C. elegans*, which were individually immobilized on magnetic beads via the His6 tag at the C terminus of these recombinant enzymes. The sequence alignments (Supplementary Fig. 1) from 69 RaPID-derived clones resulted in 11 independent sequence families, **Bm-1**–**7** and **Ce-1**–**4** obtained from panning *B. malayi* and *C. elegans* iPGMs, respectively, and corresponding to macrocyclic peptides of a lariat structure with ring sizes ranging between 7–13 amino acids and C-terminal tails of 1–7 amino acids (Table 1).

It should be noted that the cyclic peptides as isolated by RaPID are tethered at their carboxyl terminus via puromycin to the encoding mRNA (Fig. 1b). Any effect of the tethered nucleic acid during cyclic peptide binding to their target, either to facilitate binding or block possible productive target-cyclic peptide interactions is an inherent property of mRNA-display technology. Significant binding contributions made via the nucleic acid will not be present in samples made by the solid-phase peptide synthesis step.

### Functional evaluation of cyclic peptide PGM inhibitors

To efficiently profile the activity of the cyclic peptides derived from *in vitro* selection, several PGM enzymes from a range of species were evaluated, including the parasite target, *B. malayi* iPGM and filarial orthologues (*Onchocerca volvulus*, *Dirofilaria immitis*), the corresponding model organism *C. elegans* iPGM orthologue, and the *H. sapiens* anti-target dPGM isozyme. iPGM and dPGM enzymes from *Escherichia coli* were also included. We developed 1,536-well plate kinetic and end point assays for the PGM-catalysed conversion of 3-PG to 2-PG. Each assay utilized a coupled enzyme approach where the product 2-PG is driven through phosphoenol pyruvate to pyruvate and ATP via enolase and pyruvate kinase, respectively^16^. A kinetic absorbance output was achieved using lactate dehydrogenase-mediated changes in NADH concentration through pyruvate conversion to lactate (Supplementary Fig. 2a, step 3a)^17^,^18^. For the bioluminescent end point assay the ATP produced from the coupled enzymatic reactions is used by Firefly luciferase and luciferin to generate measurable light (Supplementary Fig. 2a, Step 3b; Supplementary Table 1). The continuous NADH-dependent absorbance assay was used to determine the relative activity and 3-PG *K*_M_ for each of the seven PGM orthologues and isozymes being used in this study. Each of these enzymes and conditions were used to calibrate the bioluminescent end point assay that gave a 3.5–5.4-fold ratio of signal-to-background following a five or 15 min incubation depending on the enzyme concentration (Supplementary Fig. 3; Supplementary Table 2).

For the direct evaluation of cyclic peptides on the target enzyme, we used gradient elution moving boundary electrophoresis (GEMBE)^19^,^20^ to enact an electrophoretic separation of 3-PG and 2-PG^21^ (Supplementary Figs 2b and 4). The method provides a direct, label-free measurement of the substrate and product, 3-PG and 2-PG (Supplementary Fig. 4a, top). Baseline separation of the isomers was apparent in the first derivative plot (Supplementary Fig. 4a, bottom) for equimolar amounts of 3-PG and 2-PG. Using the established separation conditions, a time course for conversion of 2-PG to 3-PG was demonstrated with the *C. elegans* and *B. malayi* iPGMs (Supplementary Fig. 4b). GEMBE while low throughput, provides ideal orthogonal validation of the activity of the iPGM inhibitors described in this study.

### Ipglycermides are potent and selective iPGM inhibitors

We initially panned the parasitic target *B. malayi* iPGM against the *N*-chloroacetyl-D-tyrosine-initiated macrocyclic peptide library through five rounds to obtain seven macrocycles represented by 11–14-mer peptides, **Bm-1**– **Bm-7** (Table 1). Enrichment is considered sufficient when sequence redundancy is observed between the final and penultimate rounds (Supplementary Fig. 1). The peptide sequences deduced from the corresponding tethered nucleic acid were chemically synthesized as cyclic peptides in sufficient quantities for evaluation as inhibitors of the enzymatic activity of a panel of seven PGM orthologues and isozymes using the bioluminescence endpoint assay (Table 1). Concentration response curves obtained for **Bm-1**−**7** across the PGM panel primarily revealed a selective, modestly potent macrocyclic series. All except **Bm-2**, (for which RaPID selection may have been influenced by the tethered nucleic acid not present in the resynthesized cyclic peptide) inhibited the iPGM orthologues in the high nanomolar to low micromolar range (Table 1), and further showed complete selectivity for iPGM versus dPGM enzymes. These cyclic peptides comprised two groups, one having large rings of 12 or 13 amino acids with short 1–2 amino-acid tails (**Bm-1**−**Bm-3**) and the second having 7-membered rings with C-terminal extensions of 4 amino acids (**Bm-4**−**Bm-7**), and all seven peptides contained a cysteine at the penultimate position. Because **Bm-4** represented the shorter, more potent class of sequences among the cyclic **Bm**-series peptides it was chosen as a template for additional study through C-terminal truncation analogues (**Bm-4a**−**Bm-4d**, Supplementary Table 3). In addition, to probe the importance of the free sulfhydryl group of the tail cysteine, Cys10 was replaced with serine. Interestingly, the Cys10Ser replacement and elimination of all but the terminal Gly11 resulted in inactive macrocycles (Supplementary Table 3).

**Figure 2:**
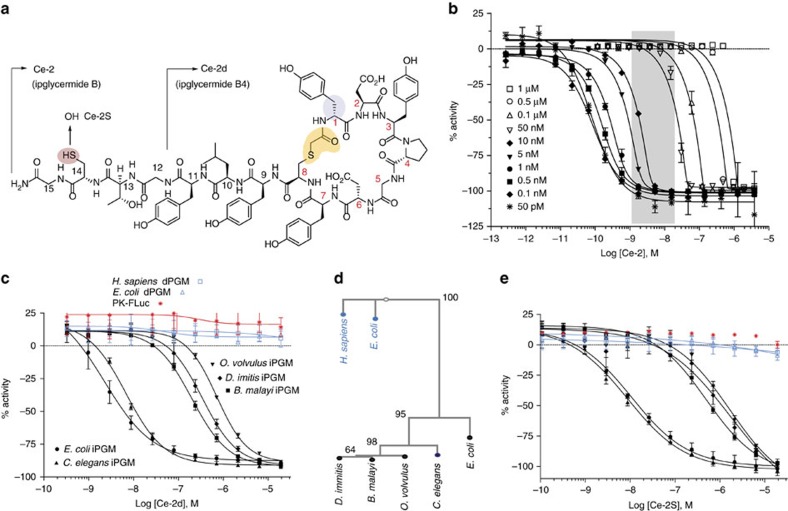
Pharmacologic–phylogenetic relationship of iPGM macrocyclic peptide inhibitor. (**a**) Structure of the **Ce-2** macrocycle obtained from affinity selection showing truncation to give **Ce-2d** and position of Cys14Ser substitution. Note the thioether bond (yellow), D-tyrosine (blue), and thiol of Cys14 (light red). (**b**) IC_50_ dependence of **Ce-2** on *C. elegans* iPGM enzyme concentration (50 pM to 1 μM), grey region indicated concentration range for PGMs used in initial inhibitory activity profiling obtained in Table 1. (**c**) Concentration-response curves for characterization of **Ce-2d** on the iPGM orthologues and dPGM isozymes. (**d**) Phylogenetic tree constructed for amino-acid sequence alignments of seven species orthologues and isozymes of PGM. Percentage bootstrap values based on 1,000 replicates are indicated at branch nodes. (**e**) Concentration-response curves for characterization of **Ce-2S** on the iPGM orthologues and dPGM isozymes. All data determined from the enzyme-coupled bioluminescent assay; PGM concentrations as indicated in Table 1. Plots are representatives from individual experiments (*N*=3); error bars are standard deviations values of technical replicates.

Our initial RaPID experiments resulted in the **Bm**-series cyclic peptides, which have relatively modest potencies. In addition *B. malayi* iPGM had proven refractory to crystallographic structure determination (*vide infra*). However, successful crystals were obtained for *C. elegans* iPGM and motivate RaPID targeting of this orthologues towards the deduction of design rules guiding macrocycle-iPGM interactions. A second set of affinity selection experiments used the *C. elegans* model organism iPGM for panning with either an *N*-chloroacetyl D- or L-tyrosine-initiated macrocyclic peptide library. Seven rounds of panning with the D-Tyr library, and six rounds with the L-Tyr library yielded two macrocyclic peptides from each library, either 15-mer peptides, ipglycermides A (**Ce-1**) and B (**Ce-2**, Fig. 2a) or 14-mer peptides (**Ce-3**, **Ce-4**), respectively. Analogous medium and large ring systems to those obtained from the *B. malayi* iPGM panning (Table 1) were isolated, but displaying some level of orthologue selectivity (for example, compare **Ce-1** with **Ce-3** in Table 1). As in the corresponding *B. malayi*-derived series ring systems the macrocycles are completely inactive toward the dPGM isozymes. Two of these, **Ce-1** and **Ce-2**, ipglycermide A and B, respectively, are exceedingly potent inhibitors of the iPGM orthologues nominally exhibiting low nM activity (2–20 nM) under the initial conditions (that is, the [iPGM] for *C. elegans, B. malayi* was 5 nM, for *D. immitis*, *E. coli* 10 nM and *O. volvulus* 20 nM) used in the endpoint profiling assays, and were recapitulated in the GEMBE-based assay for the selected PGMs shown in Supplementary Fig. 5. The steep concentration response curves observed at enzyme concentrations 5 nM and higher for **Ce-2** (Fig. 2b; Supplementary Fig. 5) suggest stoichiometric titration of the enzymes, which would occur under conditions where [E]>*K*_d_ of the ligand^22^,^23^, and therefore lead to an underestimation of potency. This is supported by the hyperbolic response of the concentration response curves and leftward shift in ipglycermide B IC_50_ as the iPGM concentration was taken below 5 nM (Fig. 2b). Using a quadratic model^22^,^24^ (Supplementary Methods) to account for stoichiometric binding at high iPGM concentration the family of concentration response curves in Fig. 2b was used to estimate an effective *K*_d_ of 73±15 pM for ipglycermide B.

### Ipglycermide core displays iPGM orthologue selectivity

To dissect the contribution of the cyclic from the linear sequence of the **Ce-2** lariat structure, and define the minimal sequence needed for activity we conducted a structure activity relationship study involving a C-terminal truncation/substitution series (Supplementary Fig. 6). Further, linear analogues were prepared to determine the effect on potency from constraining conformational states of the peptide. While removing the majority of the linear sequence of **Ce-2** resulted in loss or greatly diminished iPGM inhibitory activity (**Ce-2e**–**Ce-2g**), truncated analogues **Ce-2b**–**Ce-2d** displayed a broadened range of activity among the iPGM orthologues (Table 2). Of particular note is the shift from subnanomolar to nanomolar activity of analogues **Ce-2d** for *C. elegans* and *E. coli* iPGM, while the activity for *B. malay*i, *O. volvulus* and *D. immitis* approach IC_50_s in the micromolar range (Fig. 2c and Supplementary Fig. 7). This separation of orthologues activity closely parallels the phylogenetic differences between the amino-acid sequences of these enzymes (Fig. 2d).

### Ipglycermide subnanomolar affinity dependent on Cys14 thiol

Other than **Ce-2** only **Ce-2a**, resulting from Gly14 truncation, retains subnanomolar potency against the iPGM orthologs (Table 2) pointing to Cys14 as a key determinant in high-affinity binding. Isosteric replacement of Cys14 with Ser in **Ce-2** to generate **Ce-2S** caused an approximately 100-fold decrease in inhibitory activity (IC_50_∼10 nM) for the *C. elegans* and *E. coli* iPGMs, and comparable to **Ce-2d** a separation in potency of 100-fold between the iPGMs of *C. elegans* and *E. coli* versus *B. malayi*, *O. volvulus* and *D. immitis* (Fig. 2c,e). These results indicate that the high-affinity binding of **Ce-2** is dependent on its Cys14 thiol, possibly involving a sulfur-transition metal ion interaction at the catalytic centre of iPGM. Finally, while Cys14 contributes to important binding interactions, a peptide devoid of the macrocyclic core comprised solely of **Ce-2** residues 9–14 alone (**Ce-2tail**) is inactive on all PGMs (Table 2; Supplementary Fig. 7b).

The large entropic contribution of macrocyclization of the peptide was demonstrated by comparing the IC_50_s between **Ce-2** and a linear form of **Ce-2, Ce-L2,** made by a Cys8Ser substitution, for iPGMs on which there was measurable activity of **Ce-L2**. The most reliable data from *C. elegans*, *O. volvulus* and *E. coli* iPGM (Supplementary Fig. 7b; Table 2) allowed a calculation of between a 2,000- and 10,000-fold enhancement in affinity attributable to reduction of random states by macrocycle formation, though a potential steric clash between hydrogens replacing the thioether bond may contribute as well to this greatly decreased inhibitory activity. A related peptide derived from a linear form of **Ce-2d**, **Ce-L2d,** was completely inactive on all iPGMs, further supporting the importance of two functional lariat macrocycle sub-domains, the ring system and C terminus, both necessary to engender high-affinity binding to the **Ce-2** macrocycle.

### Structure of a nematode iPGM

The iPGMs are monomeric bi-domain enzymes where a phosphatase domain, structurally related to the alkaline phosphatase family of binuclear metalloenzymes, is connected by two hinge peptides to a phosphotransferase domain^8^. X-ray crystal structures have been obtained for the enzymes derived from two trypanosomatids and several bacterial species^8^,^25^,^26^,^27^.

To develop a structural model that delineates the molecular interactions mediating the pharmacologic–phylogenetic relationship between the macrocycles and iPGM orthologues, we attempted to co-crystallize **Ce-2** with *B. malayi* and *C. elegans* iPGM, but failed to obtain crystals. Although soaking of pre-formed iPGM crystals with **Ce-2** caused the crystals to shatter, we succeeded in obtaining two *apo* crystal forms, monoclinic *P* (iPGM-m) and orthorhombic *P* (iPGM-o) lattices (Supplementary Fig. 8a–c) of native *C. elegans* iPGM. These crystals provided the structure of a nematode iPGM (Supplementary Table 5; Supplementary Fig. 9). As anticipated from primary amino-acid sequence homology among iPGM orthologues, *C. elegans* iPGM is quite similar to other iPGMs of bacterial origin (Supplementary Fig. 10). Superposition of the monoclinic *C. elegans apo* iPGM (PDB: 5KGM) structure with *Bacillus anthracis apo* iPGM (PDB: 2IFY) yielded an root mean squared deviation (RMSD) of 2.76 Å between Cα atoms (476 residues aligned). Although the deviation from superposition is fairly large between the two structures, the overall fold is very similar (Supplementary Fig. 11). The structure of monoclinic *C. elegans* iPGM was also compared with that of a substrate bound iPGM from *Bacillus stearothermophilus* (PDB: 1O98). Superposition yielded an RMSD of 1.07 Å between Cα atoms. However, only 282 residues could be aligned for residues in the transferase domain since substrate binding produces a large conformational change in the phosphatase domain (Supplementary Fig. 11). Similar to other iPGMs, Mn^2+^ occupies one of the two phosphatase domain metal ion binding sites while, as in alkaline phosphatase, a Zn^2+^ ion was found in the second binding site of *C. elegans* iPGM. The identity of these transition metal ions was verified from phased anomalous difference maps calculated from data collected at wavelengths of 1.0000 and 1.9016 Å. These metal ions contact the histidine and aspartate triads as shown in Supplementary Fig. 12 at coordination distances listed in Supplementary Table 6, and the catalytic Ser86 nucleophile coordinates to the Zn^2+^ ion.

### Co-crystal structure elucidates inhibitory mechanism

**Figure 3:**
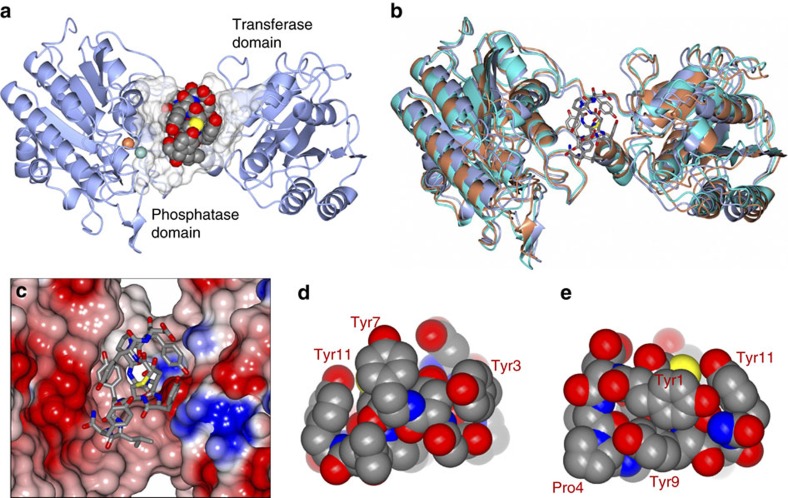
Ce-2d traps iPGM in an open conformation. (**a**) One subunit of the asymmetric unit showing the binding mode of the **Ce-2d** macrocycle to *C. elegans* iPGM. The Mn^2+^ and Zn^2+^ ions are represented as blue and tan spheres, respectively, and the bound macrocycle is drawn as CPK space-filling spheres in a cavity defined by iPGM residues within 5 Å (transparent spheres). (**b**) Superposition of *C. elegans* iPGM-o (cyan), *C. elegans* iPGM-m (tan) and *C. elegans* iPGM·**Ce-2d** (aquamarine). The **Ce-2d** peptide is represented as cylinders. (**c**) Macrocycle (cylinders) positioned within a cleft of iPGM represented as an electrostatic surface. (**d**) CPK space-filling representations of **Ce-2d** illustrating the ‘capping' orientation of the five tyrosine residues (1, 3, 7, 9 and 11) and (**e**) the edge-to-face interaction of Tyr 1 and 9. Additional residues are indicated.

**Figure 4:**
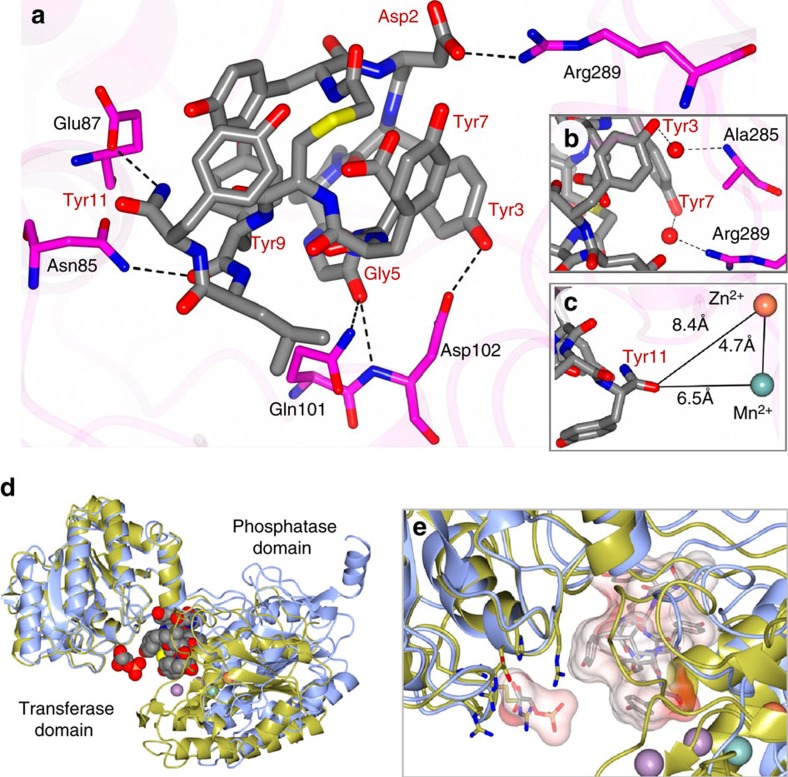
Ce-2d · iPGM interactions. (**a**) Hydrogen bond interactions (black dashed lines) between *C. elegans* iPGM and **Ce-2d**. Direct interactions and (**b**) water (red spheres) mediated contacts. (**c**) Distance from the C-terminal amide of Tyr11 and the Zn^2+^ and Mn^2+^ ion centres. (**d**) Superimposed structure of *C. elegans* iPGM·**Ce-2d** with that of *Staphylococcus aureus* iPGM in 2-phosphoglyceric acid bound form (PDB: 4NWX). The following, *S. Aureus* : *C. elegans* residue pairs were used for alignment: 123His147, 153-154Asp177-178, 191Arg216, 185Arg210, 257Arg284 and 260Arg287. **Ce-2d** and 2-PG are shown as CPK space filling models. The purple spheres are the Mn^2+^ ions of *S. aureus* iPGM and the blue and tan spheres are the Mn^2+^ and Zn^2+^ ion, respectively of *C. elegans* iPGM. (**e**) Enlarged region from d showing the relative locations of the 2-PG and **Ce-2d** as cylinder models with transparent van der Waals surfaces and alignment residue side chains clustering around 2-PG.

From the *apo* iPGM structure we observed that the N-terminal 18 amino acids, unique to *C. elegans* iPGM, were disordered. In a subsequent crystallization effort, using an 18 amino-acid N-terminal truncated form of *C. elegans* iPGM, we prepared a pre-formed **Ce-2** complex purified by sizing chromatography, as well as a mixture with **Ce-2d**. The latter resulted in needle-like crystals diffracting to 1.95 Å (Supplementary Fig. 8d and Supplementary Table 5). The final model of the iPGM·**Ce-2d** complex (PDB: 5KGN) contained two molecules in the asymmetric unit (Supplementary Fig. 13a), exhibiting an inter-domain binding mode with the macrocycle cradled in a pocket shaped from the hinge peptides and adjacent phosphatase and transferase domain surfaces (Fig. 3a). The two subunits of the asymmetric unit are nearly identical with an RMSD of 0.20 Å between Cα atoms for 520 residues aligned. Therefore all subsequent analyses were carried out using subunit A of the model. The structure of iPGM·**Ce-2d** was compared with the aforementioned *apo* iPGM-m and iPGM-o (PDB: 5KGL) structures. Superposition yielded an RMSD deviation of 1.98 Å (503 residues) and 2.05 Å (502 residues), respectively. Although the RMSD are somewhat large, the overall structures are remarkably similar (Fig. 3b) with slight displacement of secondary structure elements due to the high flexibility of iPGM. The cavity that accommodates the binding of the **Ce-2d** cyclic peptide is very similar amongst all of the structures with no marked conformational changes observed to accommodate binding of the peptide. Rather, it appears that the peptide adopts an optimal fit within this cavity as might be expected from the affinity selection approach used to discover the parent macrocycle. This region of iPGM forms a somewhat negative asymmetrically charged pocket that accommodates the polar residues of **Ce-2d** as shown in Fig. 3c. The total surface area of the **Ce-2d** peptide is 432.1 Å^2^ as calculated using Areaimol^28^, which provides information regarding total area, contact area and the solvent exposed area of a surface. A relatively small region of the total **Ce-2d** peptide surface makes direct contact to iPGM (127.1 Å^2^) with the remaining area (305 Å^2^) exposed to solvent as it is positioned within an open pocket between the phosphatase and transferase domains. On the basis of molecular weight (1501.6) and its 27 ring atoms **Ce-2d** can be categorized as a large macrocycle^29^. With 29% of its surface buried, **Ce-2d** solvent exposes slightly more surface area than comparably sized macrocylces. The electron density for the cyclic peptide was prominent for all of the residues except for the terminal tyrosine side chain which was somewhat disordered, while the C-terminal 4 residues form a short α-helix (Supplementary Fig. 13b,c). From the CPK representation of **Ce-2d** (Fig. 3d,e) the orientation of the tyrosine side chains Tyr3, Tyr7 and Tyr11 can be seen enfolding the cyclic peptide (Fig. 3d) while tyrosine side chains Tyr1 and Tyr9 engage in an edge-to-face interaction (Fig. 3e). In **Ce-1**, His7 replaces Tyr7 maintaining similar activity (Table 1). The three extra-cyclic C-terminal residues, Tyr9, Leu10 and Tyr11 are wrapped close to the core macrocycle, with the carboxamide of Tyr11 visible on the exterior surface of this compact structure and pointing toward the metal ion active site (Figs 3e and 4). Notably, the **Ce-2d** C-terminal amide of the Tyr11 residue is 6.5 and 8.4 Å from the Mn^2+^and Zn^2+^ ions, respectively (Fig. 4c). Thus, it is feasible that the longer C terminus of **Ce-2** (and **Ce-1**) could potentially extend from this cavity, positioning Cys14 within coordination distance to either metal ion.

The **Ce-2d** macrocycle forms direct hydrogen bonds with *C. elegans* iPGM as well as water-mediated contacts as depicted in Fig. 4a,b and detailed in Supplementary Table 6. Two of these key H-bonds are made with C-terminal tail residues, Tyr9 and the carboxamide of Tyr11. Others include, iPGM Arg289 which forms two H-bonds with **Ce-2d** ring system residues, one directly with Asp2 and one water-mediated through the Tyr3 hydroxyl (Fig. 4b), while a bifurcated Gly5 carbonyl H-bond occurs with iPGM via Gln101 and Asp102 (Fig. 4a). Hydrophobic interactions are also observed. For example, Leu10 of the macrocycle sits in a small pocket formed by iPGM Ile103, Leu78 and Leu82 (Supplementary Fig. 14), while Ile99 of the enzyme is within 3.5 Å or less of cyclic peptide residues Tyr9, Leu10 and Tyr7.

To explore the orientation of **Ce-2d** binding relative to phosphoglycerate, we superimposed the *S. aureus* iPGM 2-PG-bound^30^ and *C. elegans* iPGM·**Ce-2d** crystal structures using the His, Asp, Arg phosphoglycerate binding residues as alignment points in both structures (Fig. 4d,e). The model places the macrocycle at a site non-overlapping with phosphoglycerate supporting an allosteric binding mode for the ipglycermides. This result is consistent with the independence of **Ce-2d** and **Ce-2** IC_50_ on 3-PG substrate concentration (Supplementary Fig. 15).

**Figure 5:**
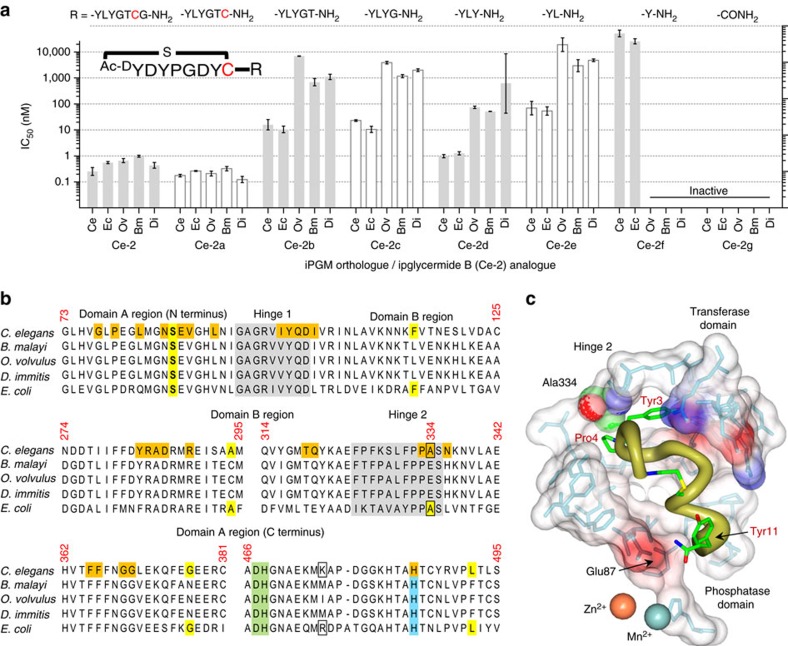
Structural basis underlying the pharmacologic–phylogenetic Ce-2 macrocycle series-iPGM orthologue relationship. (**a**) Relationship between **Ce-2** macrocycle truncation series IC_50_s and iPGM orthologues. Analogues with no detectable inhibitory activity are indicated as inactive. Data represent mean±s.d. values of the log normal distributed IC_50_s determined for the given peptide for experiments with ≥4 replicates; otherwise error bar is determined from the nonlinear fit of the standard Hill equation to the aggregated data from the replicates. Values are from Supplementary Table 3 converted from pIC_50_ where IC_50_=10^-pIC_50_^. (**b**) Select amino-acid sequence alignment of iPGM orthologues in this study (see Supplementary Fig. 10 for full alignment). iPGM residues within 5 Å of **Ce-2d** are coloured orange. Residues identical between *C. elegans* and *E. coli* iPGM are coloured yellow; grey indicated hinge regions; green and blue are amino acids that ligand metal ions. (**c**) Cavity formed from *C. elegans* iPGM residues (light blue chain under transparent spheres) within 5 Å of the **Ce-2d** macrocycle shown as a worm α-chain (gold) representation scaled by B-factor with select side chains (Tyr3, Pro4, thioether linkage, and C-terminal Tyr11 amide) shown. The iPGM Ala334 residue is shown as a CPK space fill. Electrostatic surface of the **Ce-2d** binding cavity is also shown.

To gain insight into the mechanism underlying the iPGM orthologue selectivity of the **Ce-2** series observed in Fig. 5a, we began by defining a binding cavity from residues within 5 Å of the **Ce-2d** macrocycle (shaded orange in the protein sequence alignment shown in Fig. 5b). In the cavity defined by these amino acids we projected **Ce-2d** as a worm representation α-chain scaled by *B*-factor (gold) with several side chains, Tyr3, Pro4, Tyr11 amide and the thioether linkage shown in green (Fig. 5c), from which several salient observations can be made. As previously discussed, truncation of **Ce-2** beyond Cys14 results in a >10-fold potency decrease (**Ce-2b**, **c**) until Tyr11 becomes the C-terminal residue, at which point potency for *C. elegans* and *E. coli* iPGM is recovered (**Ce-2d**), but only marginally so for the *B. malayi*, *O. volvulus* and *D. immitis* orthologues. The improvement in potency is likely the result of a new H-bond made possible by the C-terminal Tyr11 amide of **Ce-2d** with the highly conserved Glu87 of the phosphatase domain (Fig. 5c). Subsequent removal of Tyr11 (**Ce-2e**) results in nearly a 100-fold potency decrease, probably a consequence of the Tyr11 amide—Glu87 H-bond forfeiture. Continued truncation leads to virtual inactivation of the macrocycle (**Ce-2f**,**g**). A possible explanation for the dramatic separation of **Ce-2d** inhibitory potency between *C. elegans* and *E. coli* versus *B. malayi*, *O. volvulus* and *D. immitis* iPGM orthologues may be in part mediated by Ala334 within hinge 2 of *C. elegans* and *E. coli* iPGM, but replaced by a glutamate in the *B. malayi*, *O. volvulus* and *D. immitis* iPGM orthologues. Ala334 is <2.5 Å from **Ce-2d** Pro4 and Tyr3, thus the larger volume occupied by a glutamic acid residue may create a steric clash only partially compensated for by the Tyr11 amide H-bond. Sequence differences outside the binding cavity between *C. elegans* and *E. coli* versus the *B. malayi*, *O. volvulus* and *D. immitis* iPGMs highlighted in yellow in Supplementary Fig. 10 could also contribute to orthologue selectivity.

## Discussion

The glycolytic phosphoglycerate mutase, iPGM is an attractive target for the development of broad spectrum antiparasitic and antibacterial agents owing to its essential metabolic function and evolutionary divergence from the human enzyme^10^,^25^. Infectious organisms potentially susceptible to an iPGM inhibitor are responsible for diseases ranging from African trypanosomiasis (sleeping sickness), lymphatic filariasis (elephantiasis tropica), onchocerciasis (river blindness), *Staphylococcus aureus* toxic shock syndrome and *Bacillus anthracis* intoxication. Difficulty in achieving small-molecule inhibitors of even modest potency toward iPGM may be rationalized by our current understanding of the catalytic mechanism in which a fully formed, solvent-inaccessible active site assembles dynamically upon phosphoglycerate binding^30^. Identification of only low-potency metal ion chelators from small-molecule HTS is not unexpected given the absence of a hydrophobic druggable pocket and dependence of enzyme activity on transition metal ions^12^. Resembling a protein–protein interaction, the charged, dynamic domain motions observed in iPGM catalysis necessitate the exploration of new chemical space that can present protein surface complementarity to achieve an inhibition mechanism^13^.

In an effort to elucidate additional ligand interfaces of iPGMs for the study of phosphoglycerate mutase enzymology and inhibition thereof, we applied an affinity selection approach to address the limited protein surface topology of small-molecule chemical libraries. As a complementary methodology to HTS-based ligand discovery^31^, *in vitro* affinity display technologies generating peptide^32^,^33^, DNA or RNA^34^ ligands can expose currently ‘undruggable' target space by defining new molecular topologies to inform the design of complementary ligand scaffolds in synthetic small molecules. To this end, we pursued macrocyclic polypeptide templates incorporating rigid stereochemical complexity extensively used by nature to interact across extended protein binding surfaces^29^,^35^. Macromolecular diversity of the cyclic peptide library used in this study exceeds by several million-fold that achieved thus far from small-molecule HTS. The self-encoded nature of this cyclic peptide repertoire enables the amplification of low-abundance, high-affinity ligand subpopulations selected using an immobilized iPGM subtype. Candidate ligand sequences obtained from iterative rounds of enrichment were resynthesized by solid-phase peptide synthesis (SPPS) yielding potent and selective iPGM inhibitors (Fig. 1). Our co-crystal structure reveals that **Ce-2** stabilizes a locked-open structure, which precludes the dynamic rearrangement of domains required for catalysis. Such a dynamic-constraint inhibition mechanism is an important benefit of cyclic peptide libraries owing to their larger size, which can span greater distances between flexible protein domains.

**Figure 6:**
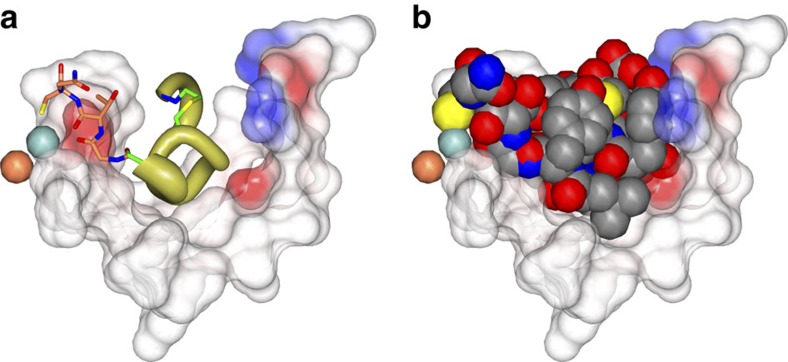
Modelling of C-terminal residues of Ce-2 onto Ce-2d. (**a**) The **Ce-2d** macrocycle is shown as worm α-chain (gold) representation scaled by B-factor within a cavity of *C. elegans* iPGM residues (transparent spheres) formed from residues within 5Å of cyclic peptide. The C-terminal residues, -Gly12-Thr13-Cys14-Gly15 of **Ce-2** were modelled onto the iPGM·**Ce-2d** complex and are shown as tan sticks extending from **Ce-2d**. Electrostatic surface of the binding cavity is also shown. (**b**) **Ce-2** van der Waals radii shown using a CPK model. The Cys14 sulfhydryl is shown in yellow.

SPPS of active cyclic peptide compounds facilitates rapid examination of the molecular details of the inhibitory mechanism and immediately presents a synthetic path towards generating an improved second-generation inhibitor. Progressive C-terminal truncation of ipglycermide B (**Ce-2**), or introduction of a Cys14Ser point mutation, revealed that the Cys14 sulfhydryl engendered the pan-orthologue subnanomolar potency to the macrocycle. This observation is consistent with a cysteinyl thiolate functioning as a potential catalytic-site metal ion ligand, consistent with a borderline soft Lewis acid Zn^2+^ at the iPGM active site suggested by our crystallographic findings. Loss of the metal ion-anchoring sulfhydryl side chain as a consequence of truncation or Cys14Ser substitution results in potency discrimination among the iPGM orthologues (Supplementary Fig. 7a) corresponding to their phylogenetic relationship. Taken together these results suggest a two-site allosteric binding mechanism whereby the cyclic sequence and pendant C-terminal cysteine bind at distinct iPGM regions. The binding orientation of the macrocycle positions the Cys14 sulfhydryl within coordinating distance to the metal ion site (Fig. 6). An interaction between Cys14 and the Zn^2+^ ion would likely require a conformational adjustment in the enzyme and possibly explain the mechanism by which **Ce-2** stabilizes a locked-open structure.

Interestingly, the naturally occurring mono- and bicyclic depsipeptide histone deacetylase inhibitors harbour latent thiolates either as a thioester (largazole), or as a disulfide forming a second ring system (romidepsin, thailandepsin and spiruchostatin) which, upon hydrolysis or reduction, respectively, liberates an active site Zn^2+^-coordinating thiolate in mammalian cells^36^. In striking parallel to our present observation of Cys14 dependence on potency and Hill slope gradient, Wang, *et al*.^37^ demonstrate a similar correlation between the potency and Hill slopes of the reduced and oxidized forms of the bicyclic depsipeptide thailandepsin. The macrocyclic library therefore appears to encode sufficient diversity to enable a synthetic selection capable of capturing similar mechanistic and pharmacologic behaviours as the natural products that have evolved to potently and selectively target zinc-dependent histone deacetylases.

The discovery of ipglycermides represents potent isozyme-selective iPGM inhibitors to enable mechanistic and structural studies of glycolytic mutases from microorganisms. With antibody-like affinity and selectivity, though lacking *in vivo* efficacy (Supplementary Fig. 16), the ipglycermides exemplify the fertile yet uncultivated landscape between small molecules and protein biologics. The macrocyclic peptides and accompanying crystallographic information presented here for an enzyme previously considered ‘undruggable' reveal an important binding mode and inhibition mechanism that may be applicable to other difficult targets and extendable to modulation of protein–protein interactions. The interfacial contacts between ipglycermide and iPGM may inspire peptide mimetic analogues, particularly as significant regions of the macrocycle act as a scaffold to position a subset of key residues (Fig. 4). We anticipate the molecular tools and atomic-level structural guidance provided should stimulate further progress in this area.

## Methods

### Preparation of PGM enzymes

All PGM enzymes were cloned into pET21a(+) and the inserts fully sequenced to validate authenticity. Proteins were expressed in the *E. coli* strain C2566/*T7 Express (fhuA2 lacZ::T7 gene1 [lon] ompT gal sulA11 R(mcr-73::miniTn10--TetS)2[dcm] R(zgb-210::Tn10--TetS) endA1 Δ(mcrC-mrr)114::IS10*) (New England Biolabs) and expressed and purified as previously described^6^,^38^. Briefly, optimum conditions for production of soluble recombinant iPGM involved growth of cultures at 37°C to 0.6_OD600_, induction with 0.1 mM IPTG overnight at 16 °C. The His-tagged proteins were purified on a 5 ml HiTrap chelating HP column (GE Healthcare; Pittsburg, PA) using an AKTA FPLC. After application of the sample, the column was washed with five column volumes of buffer A (20 mM NaPO_4_, 300 mM NaCl, 10 mM imidazole, pH 7.4) followed by 10 column volumes of 92% buffer A:8% buffer B (20 mM NaPO_4_, 300 mM NaCl, 400 mM imidazole, pH 7.4). Protein was then eluted using a linear gradient (8–100%) of buffer B equivalent to 40–400 mM imidazole. Fractions containing iPGM-His6X were pooled, dialysed against dialysis buffer (40 mM Tris-HCl, 200 mM NaCl and 50% glycerol, pH 7.5) and stored at −20°C. Before use samples were prepared for experiments by size exclusion chromatography to separate active enzyme from aggregates formed during storage. Proteins were analysed by SDS–PAGE to confirm the predicted size and purity (Supplementary Fig. 3), and concentrations determined using the BCA protein assay. The sequences encoding the PGMs used in this study have the following NCBI reference numbers: *C. elegans* iPGM, long form, NP_871851.1; *C. elegans* iPGM, short form, NP_491896.1; *B. malayi* iPGM AAQ97626.1; *O. volvulus* iPGM, AAV33247.1; *Dirofilaria immitis* iPGM, AEA91534.1; *Homo sapiens* dPGM, NP_002620.1; *E. coli* iPGM, P37689.1; and *E. coli* dPGM, P62707.2.

### iPGM and dPGM assays

Phosphoglycerate mutase activity was measured either as a continuous (kinetic) or end point output assay (Supplementary Fig. 2). Briefly, 4 μl enzymes were dispensed into black clear-bottom 1536-well plates (Cat# 789092-F, Greiner Bio-One North America) in a pH 8.0 assay buffer with the BioRaptor FRD (Beckman Coulter) followed by 2 μl of 3-phosphoglycerate in a coupled enzyme assay buffer (includes enolase and pyruvate kinase). For the continuous assay lactate dehydrogenase was included in the coupled enzyme assay buffer, and A340 nm was recorded on an Infinite M1000 PRO (Tecan Group Ltd). For bioluminescent outputs 4 μl Kinase-Glo Plus reagent (Promega Corporation, Madison, WI) was added after a 5 min reaction, plates were incubated at room temperature for 10 min then measured by a ViewLux plate reader (PerkinElmer, Waltham, MA). Peptide solutions (5 mM stock in DMSO) were serial diluted and added to the assay plate with a Pin tool (Waco Inc.). Concentration response curves were fit using Prism (GraphPad Software, San Diego, CA). Additional details are available in Supplementary Information; Supplementary Tables 1 and 2.

### Gradient elution moving boundary electrophoresis (GEMBE)

GEMBE was used for the direct monitoring of enzyme activity via label-free measurement of the substrate and product, 2-PG and 3-PG. Enzyme reaction mixtures were prepared in the GEMBE apparatus^39^ with cyclic peptide concentrations between 195 pM and 2.5 μM for compounds **Ce-2** and **Ce-2d** (plus no inhibitor controls). Separations were performed in 5 cm long, 15 μm inner diameter capillaries with 400 V cm^−1^ electric field strength and 12.5 Pa s^−1^ pressure ramp rate. For each sample, the GEMBE separation was repeated multiple times to monitor the conversion of substrate to product. Electropherogram data (Supplementary Fig. 4) was fit to a sum of complementary error functions for quantitation. The reaction rate was determined by a linear fit to the per cent conversion versus reaction time data. Full details are available in Supplementary Information.

### Macrocyclic library design

Two thioether-macrocyclic peptide libraries were constructed with either *N*-(2-chloroacetyl)-L-tyrosine (ClAc^L^Y) or *N*-(2-chloroacetyl)-D-tyrosine (ClAc^D^Y) as an initiator by using the Flexible *in vitro* Translation (FIT) system^40^. The corresponding mRNA library is designed to have an AUG (ClAc^L/D^Y) initiator codon followed by 4–12 NNK random codons (N=G, C, A or U; K=G or U), which code random proteinogenic amino-acid residues, followed by a fixed UGC codon that assigns Cys. The theoretical diversity of the macrocycles based on the quantitative assessment of efficiencies of the individual transformation steps (see below) is at least 10^12^. After *in vitro* translation, a thioether bond formed spontaneously between the N-terminal ClAc group of the initiator ^L/D^Tyr residue and the sulfhydryl group of a downstream Cys residue.

### Affinity selection and enrichment

Affinity selections were independently performed with ^D^Y library against *B. malayi* iPGM (His_10_-tagged) and ^D/L^Y libraries against *C. elegans* iPGM (His_10_-tagged) by employing the RaPID system^41^. The mRNA libraries, ClAc-L-Tyr-tRNA^fMet^_CAU_ and ClAc-D-Tyr-tRNA^fMet^_CAU_ were prepared as reported^42^,^43^. 40 μl (2 μl from round 2) of 10 μM mRNA libraries were ligated with 80 μl (4 μl from second round) of 7.5 μM of the puromycin linker of which DNA sequence was complementary to the 3′-end constant region of the mRNA libraries using a T4 RNA ligase at 25 °C for 30 min. After purification by phenol–chloroform extraction and ethanol precipitation, 30 μl of 6 μM (from round 2, 1.5 μl of 5 μM) mRNA-puromycin conjugate and 30 μl (from round 2, 0.7 μl) of 250 μM ClAc-L-Tyr-tRNA^fMet^_CAU_ or ClAc-D-Tyr-tRNA^fMet^_CAU_ were used in a methionine-deficient FIT system to generate respective peptide libraries. Then, the *in vitro* translation reactions were performed. A solution of 30 μl of 6 μM (from round 2, 1.5 μl of 5 μM) mRNA-puromycin conjugate and 30 μl (from round 2, 0.7 μl) of 250 μM ClAc-L-Tyr-tRNA^fMet^_CAU_ or ClAc-D-Tyr-tRNA^fMet^_CAU_ were incubated at 37 °C for 30 min with an extra incubation at 25 °C. After an addition of 15 μl of 200 mM EDTA solution, the reaction solution was incubated at 37 °C for 30 min to facilitate macrocyclization and subject to pre-washed Sephadex G-25 columns to remove salts. The desalted solution of peptide–mRNA (peptide–mRNA/cDNA from round 2, *vide infra*) was applied to Dynabeads His-tag Isolation & Pulldown magnetic beads (Invitrogen) to remove undesired bead binders. This process is called pre-clearance or negative selection and was repeated twice (six times from round 2). After the pre-clearance, the peptide–mRNA (peptide–mRNA/cDNA from round 2) solution was incubated with (*B. malayi* iPGM or *C. elegans* iPGM)-immobilized Dynabeads for 30 min at 4 °C to obtain iPGM-binders. This process is referred to as positive selection. The selected fused peptide–mRNA on the beads was reverse transcribed by M-MLV reverse transcriptase (Promega) for 1 h at 42 °C. The fused peptide–mRNA/cDNA was isolated from the beads by incubating in 1 × PCR reaction buffer heated for 5 min at 95 °C. The amount of eluted cDNAs was measured by quantitative PCR. The remaining cDNAs were amplified by PCR, purified and transcribed into mRNAs as a library for the next round of selection. The library preparation, pre-clearance and positive selection were one round of the enrichment processes. Beginning with round 2 the library was reversed transcribed by M-MLV before the incubation with target protein. A significant enrichment of cDNAs was observed at the sixth round and seventh round for *B. malayi* iPGM and *C. elegans* iPGM, respectively. The recovered cDNAs were ligated into the pGEM-T-Easy vector (Promega), using TA-cloning. The vectors were transformed into DH5α competent cells; individual clones were picked and sequenced (Supplementary Fig. 1).

### Chemical synthesis of macrocycles and analogues

Macrocycles were chemically synthesized using a Syro Wave automated peptide synthesizer (Biotage) by Fmoc solid-Phase peptide synthesis (SPPS) as previously described (see Supplementary Information for additional details)^44^,^45^. Briefly, the chloroacetyl group or acetyl group was coupled onto the N-terminal amide group for the formation of cyclic or linear peptide analogues respectively after the automated synthesis. Peptides are cleaved by a solution of 92.5% trifluoroacetic acid (TFA), 2.5% water, 2.5% triisopropylsilane and 2.5% ethanedithiol and precipitated by diethyl ether. To conduct the cyclization reaction, the peptide pellet was dissolved in 10 ml DMSO/0.1% TFA in water (1:1), adjusted to pH>8 by addition of triethylamine and incubated for 1 h at 25 °C. This cyclization reaction was quenched by addition of TFA to acidify the peptide suspensions. Then peptides were purified by reverse-phase HPLC (RP-HPLC), molecular masses were verified by MALDI-TOF mass spectrometry, using a microflex or autoflex instrument (Bruker Daltonics; Supplementary Fig. 17; Supplementary Table 4).

### Macrocyclic peptide characterization across PGM orthologues

Macrocyclic peptide solutions were prepared in DMSO at a concentration beginning at 1 or 5 mM and titrated as an 11-point 1:3, or 16-point 1:2 series. For the 11-point titration series, compound dispense plates were prepared by NCATS compound management in 1536-well polypropylene deep well, v-bottom plates (Greiner Bio-One, #782270) in a single interweaved row-wise pattern per macrocyclic peptide resulting in a concentration range of 5 mM to 84.7 nM. For the 16-point titration series, compound dispense plates were prepared by hand down a single column per peptide of 384-well polypropylene deep well, v-bottom plates (Greiner Bio-One, #781270), and transferred to 1536-well polypropylene deep well, v-bottom plates with a multichannel pipette in duplicate for a concentration range of 5 mM to 152.6 nM or 1 mM to 30.5 nM. Each macrocyclic peptide was characterized across five iPGM orthologues, two dPGM isozymes and the PK-FLuc control in the Kinase-Glo Plus coupled enzyme assay described above. For the assay, 23 nl of the peptide titration series from either the 11-point or 16-point compound dispense plate were simultaneously transferred to 1536-well assay plates (Cat# 789092-F, Greiner Bio-One North America) using a 1536-pin tool (Wako) for a final concentration range of 19.2 μM–0.33 nM, 19.2 μM–0.58 nM, or 383 μM–117 pM, respectively.

*Curve fitting*. Concentration response curves reported were generated using GraphPad Prism 5 employing the sigmoidal dose-response (variable slope) curve fitting function (equation (1)):


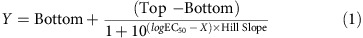


*K*_d_ from enzyme titration experiments were determined using GraphPad Prism by fitting the data series to the quadratic function^22^,^23^,^24^:


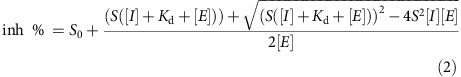


Where inh % is the apparent inhibition of the system with maximal response magnitude *S*, [*I*] and [*E*] are the inhibitor and enzyme concentrations, respectively, and *S*_0_ is an offset term to accommodate variations in the normalized signal of the assay in the absence of inhibitor. The fit of the series of titrations allows *S*, and *S*_0_ to vary for each individual titration at enzyme concentration [*E*], but *K*_d_ is a shared variable for all titrations within the series. Ranges for [*E*] and [*I*] are described in Supplementary Information.

*Error analysis*. The concentration of cyclic peptide or cyclic peptide analogues resulting in an inhibition of 50% of the indicated PGM activity tested are reported as pIC_50_ values in Supplementary Table 3. The number of independent experiments is indicated in Supplementary Table 3. Inactive peptides were tested once. All experiments with reported s.d. for error bars in Fig. 2 were conducted with two technical replicates and are representative plots from *N*≥3 independent experiments. The data used to construct Fig. 5a was from Supplementary Table 3 converted from pIC_50_ where IC_50_=10^-pIC_50_^. Error bars represent the s.d. values of the log normal distributed IC_50_s determined for the given peptide, such that 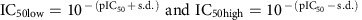.

*Exclusion criteria*. A data point would be eliminated from the curve fit if the value was determined to be an outlier based on the criteria described in Southall *et al*.^46^ Potential reasons for a data point to be eliminated from a curve fit would include, for example, known failure of compound transfer or under dispensing of assay reagent to the test well of the 1536-well assay plate. No data points needed to be excluded in the concentration response curves presented in this study.

### Phylogenetic tree construction

The protein sequences for the seven PGM orthologues (see above) were aligned using Clustal Omega (www.ebi.ac.uk/Tools/msa/clustalo/) multiple sequence alignment analysis. The Pearson/FASTA alignment was uploaded to RAxML BlackBox (www.genome.jp/tools/raxml) for tree construction^47^. Gamma model of rate of heterogeneity and the BLOSUM62 protein substitution matrix with a maximum likelihood search were applied for tree building. No outgroup was selected for tree rooting. A rapid algorithm bootstrapping analysis was performed with 1,000 replicates.

### Crystallization and data collection

Purified full length apo iPGM from *Caenorhabditis elegans* spanning and harbouring a C-terminal hexahistidine tag was concentrated to 11.6 mg ml^−1^ in 200 mM NaCl, 20 mM Tris pH 7.5, 2 mM TCEP. Another sample of iPGM from *Caenorhabditis elegans* spanning residues M19 to I539, for preparation of the peptide complex, was concentrated to 10.8 mg ml^−1^ in 150 mM NaCl, 30 mM Tris pH 8.0 for crystallization screening. To prepare the **Ce-2d** cyclic peptide complex a 50 mM stock solution of peptide **Ce-2d** was prepared in DMSO, mixed in a 1:1.5 (protein:cyclic peptide) molar ratio and incubated on ice for 30 min before screening. All crystallization experiments were conducted in Compact 300 (Rigaku Reagents) sitting drop vapour diffusion plates at 20 °C using equal volumes of protein and crystallization solution equilibrated against 75 μl of the latter.

*Apo iPGM*. Native *C. elegans* iPGM yielded crystals that formed plate clusters, which represented two crystal forms. Monoclinic *P* crystals (*C. elegans* iPGM-m) were obtained in approximately 4 weeks (Supplementary Fig. 8a) from the Wizard 3–4 screen (Rigaku Reagents) condition D11 (30% (w/v) PEG 5000 MME, 100 mM MES pH 6.5, 200 mM ammonium sulfate). The sample was subjected to refinement screening using the Additive Screen HT (Hampton Research). After ∼2 weeks, single plate shape crystals (Supplementary Fig. 8b) were observed from a condition consisting of 30% (w/v) PEG 5000 MME, 100 mM MES pH 6.5, 200 mM ammonium sulfate, 100 mM guanidine-HCl. A second crystal form belonging to an orthorhombic *P* lattice (*C. elegans* iPGM-o) was observed after 6 months (Supplementary Fig. 8c) from the Index HT (Hampton Research) condition F7 (25% (w/v) PEG 3350, 100 mM Bis-Tris pH 6.5, 200 mM ammonium sulfate). Samples were transferred to a fresh drop composed of 75% crystallization solution and 25% PEG 400 and stored in liquid nitrogen.

*iPGM ·**Ce-2d** complex*. *C. elegans* Met19 iPGM prepared in a 1:1.5 ratio with **Ce-2d** yielded crystals displaying a needle morphology after 7 days (Supplementary Fig. 8d) from the Crystal Screen HT (Hampton Research) condition A6 (30% (w/v) PEG 4000, 100 mM Tris pH 8.5, 200 mM MgCl_2_. Samples were transferred to a fresh drop composed of 80% crystallization solution and 20% glycerol and stored in liquid nitrogen.

### Structure solution and refinement

X-ray diffraction data were collected at a temperature of 100 K and a wavelength of 1.0000 Å at the Advanced Photon Source beamline 17-ID using a Dectris Pilatus 6M pixel array detector. Intensities were integrated using XDS^48^,^49^ via Autoproc^50^ and the Laue class analysis and data scaling were performed with Aimless^51^ which suggested that the highest probability Laue class was 2/*m* for iPGM-m and *mmm* for iPGM-o. The Matthews' coefficient^52^ indicated that there were two molecules in the asymmetric unit (*V*_m_=2.7 Å^3^/Da, % solvent=54%) and (*V*_m_=2.5 Å^3^/Da, % solvent=50%) for *C. elegans* iPGM-m and *mmm* iPGM-o respectively. Structure solution for iPGM-m was conducted by molecular replacement with Balbes^53^ which generated a search model using a previously determined iPGM structure (PDB: 1O98 (ref. 54)). Searches were conducted in space groups *P*2 and *P*2_1_ and the top solution was obtained in the latter space group which was used from this point forward. Initial refinement of the model with Refmac^55^ converged at *R*/*R*_free_ of 34%/37%. For iPGM-o, molecular replacement was conducted using Phaser^56^ in all possible space groups with 222 point symmetry using PDB 2IFY^27^ as the search model. The top solution was obtained in the space group *P*2_1_2_1_2_1_. The models were improved by automated model building with Phenix^57^.

Structure solution for iPGM ·**Ce-2d** was conducted by molecular replacement using a single subunit of iPGM-o as the search model. Searches were conducted in space groups *P*2 and *P*2_1_ (*V*_m_=2.3 Å^3^/Da, % solvent=47%) for two molecules in the asymmetric unit and the top solution was obtained in *P*2, which was used from this point forward. Initial refinement of the model was carried out with Refmac^55^ and was improved by automated model building with Apr/warp^58^. Subsequent refinement and manual model building were carried out with Phenix and Coot^59^, respectively. Disordered side chains were truncated to the point for which electron density could be observed. Structure validation was conducted with Molprobity^60^ and figures were prepared using the CCP4MG package^61^. Superposition of iPGM structures was conducted using GESAMT^62^ via the CCP4 (ref. 63) interface. The following Ramachandran plot statistics were obtained for the final models. *C. elegans* iPGM- m (favoured: 97.6%, allowed: 1.8%, outliers: 0.6%), *C. elegans* iPGM- o (favoured: 96.6%, allowed: 3.1%, outliers: 0.3%) and *C. elegans* iPGM-Ce-D2 (favoured: 98.0%, allowed: 1.7%, outliers: 0.3%). Relevant crystallographic data are provided in Supplementary Table 5.

### Crystallographic analysis

The final model of iPGM-m consisted of two subunits with two Mn^2+^ and Zn^2+^ ions modelled within domain A of each subunit (Supplementary Fig. 9a) and the first 20 residues of the N terminus and last 13 residues of the C terminus were disordered and could not be modelled. The two subunits are nearly identical with an RMSD deviation of 0.58 Å between Cα atoms for 517 residues aligned using GESAMT^62^ (Supplementary Fig. 9b). Crystals of the orthorhombic form (*C. elegans* iPGM-o) were obtained after approximately 6 months and diffracted to higher resolution than iPGM-m. Similarly, the N- and C-terminal residues were disordered in the iPGM-o as well.

*Metal ion sites*. Large peaks of positive electron density (Fo–Fc) were observed in the metal binding sites of the phosphatase domain following refinement Supplementary Fig. 12a. This region is occupied by Asp 426, His 430 and His 485 (site1) and Asp 37, Ser 86, Asp 467 and His 468 (site2). On the basis of the coordination distances and electron density (difference and anomalous), Mn^2+^ and Zn^2+^ ions were assigned at these respective sites. Further details are provided in the Supplementary Information.

### Data availability

Coordinates and structure factors have been deposited to the Protein Databank with the following accession codes: *C. elegans apo* iPGM-m (5KGM), *C. elegans apo* iPGM-o (5KGL) and the complex *C. elegans* Met19 iPGM · **Ce-2d** (5KGN). The data that support the findings of this study are available from the corresponding author upon request.

## Additional information

**How to cite this article:** Yu, H. *et al*. Macrocycle peptides delineate locked-open inhibition mechanism for microorganism phosphoglycerate mutases. *Nat. Commun.* **8**, 14932 doi: 10.1038/ncomms14932 (2017).

**Publisher's note:** Springer Nature remains neutral with regard to jurisdictional claims in published maps and institutional affiliations.

## Supplementary Material

Supplementary Information Supplementary Figures, Supplementary Tables, Supplementary Methods and Supplementary References

## Figures and Tables

**Table 1:**
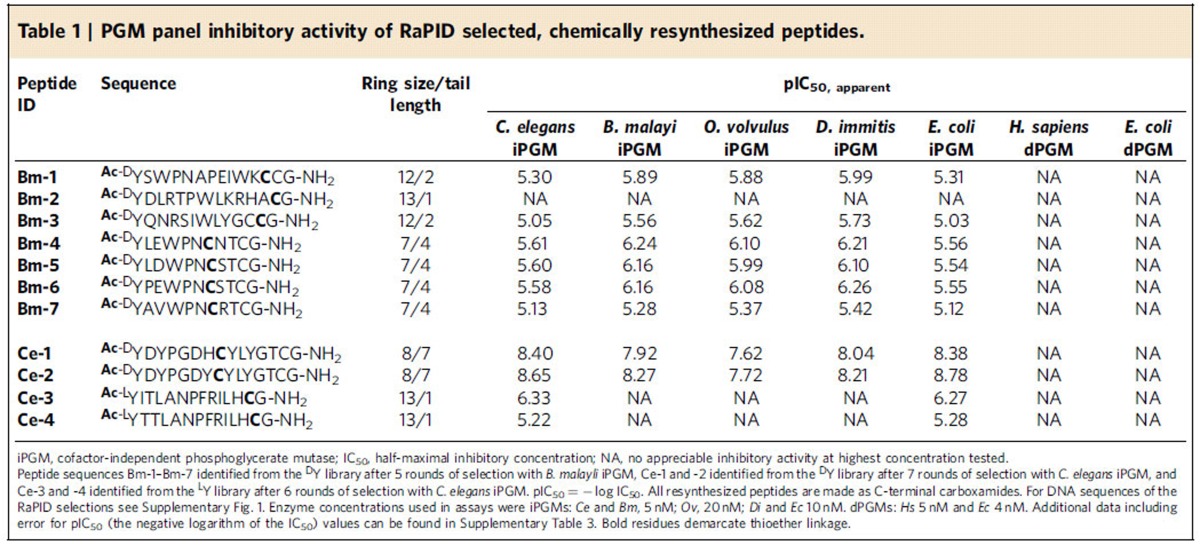
PGM panel inhibitory activity of RaPID selected, chemically resynthesized peptides.

**Table 2:**
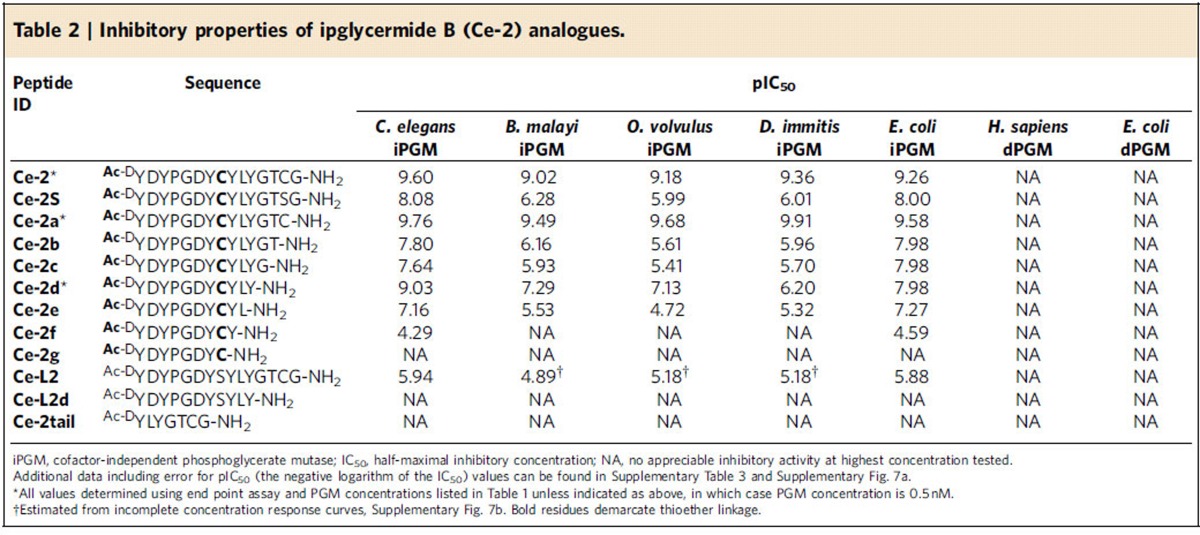
Inhibitory properties of ipglycermide B (**Ce-2**) analogues.
