# Supplementary material for: Macrocycle peptides delineate locked-open inhibition mechanism for microorganism phosphoglycerate mutases
Source: Nat Commun. 2017 Apr 3;8:14932. doi: 10.1038/ncomms14932 (PMC5382265; doi:10.1038/ncomms14932)
Supplement: Supplementary Information — Supplementary Figures, Supplementary Tables, Supplementary Methods and Supplementary References [file ncomms14932-s1.pdf]

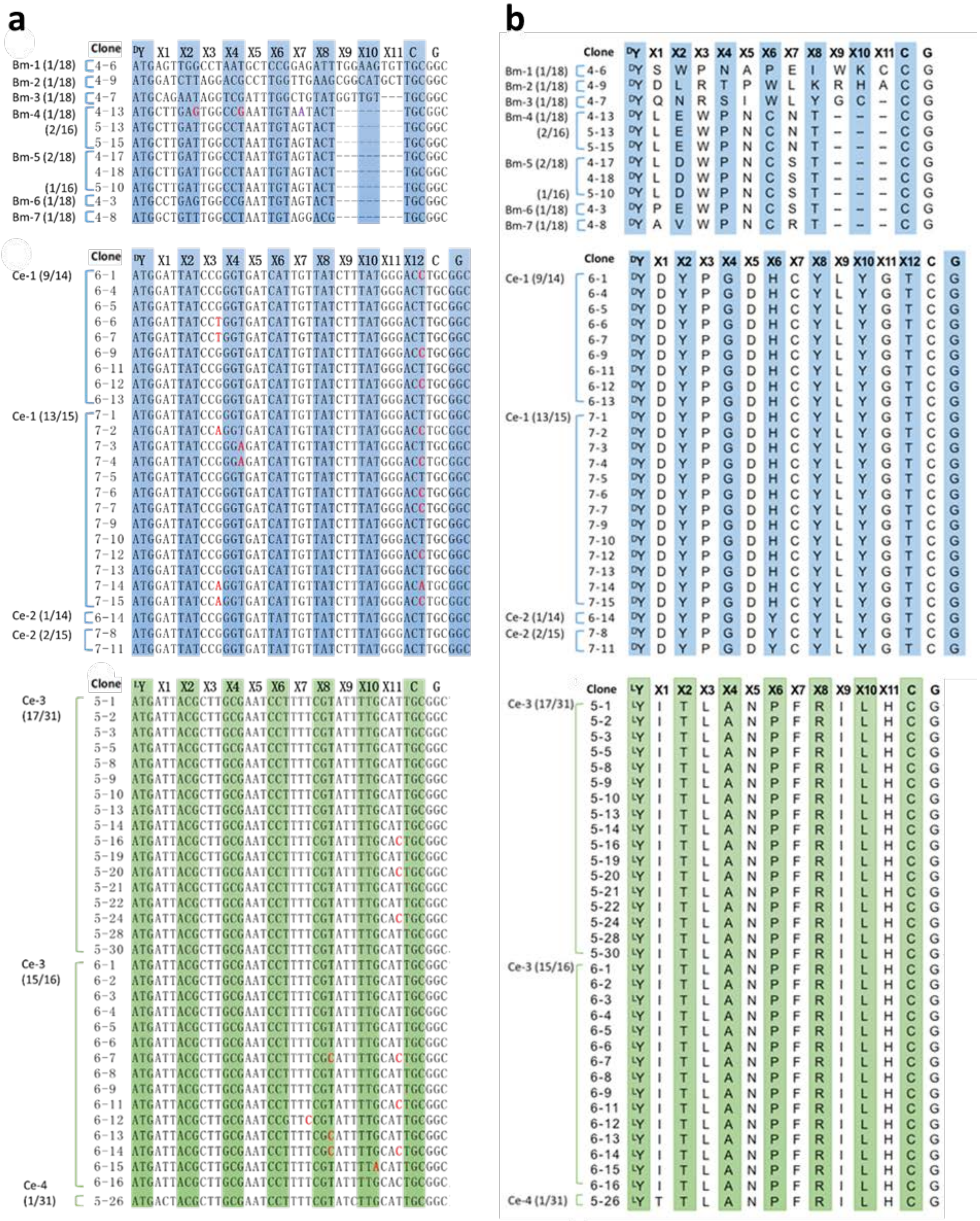

Supplementary Figure 1. *DNA sequence (**a**) and corresponding (**b**) peptide sequence alignments of the RaPID selections.* (*top*) Alignment of DNA sequences and peptide sequences identified from 4th and 5th rounds of selection with ᴰY library against *B. malayi* iPGM. (*middle*) Alignment of DNA sequences and peptide sequences identified from 6th and 7th rounds of selection with ᴰY library against *C. elegans* iPGM. (*bottom*) Alignment of DNA sequences and peptide sequences identified from 5th and 6th rounds of selection with ᴸY library against *C. elegans* iPGM. Mutations at second or third codon base are colored in purple or red, respectively.

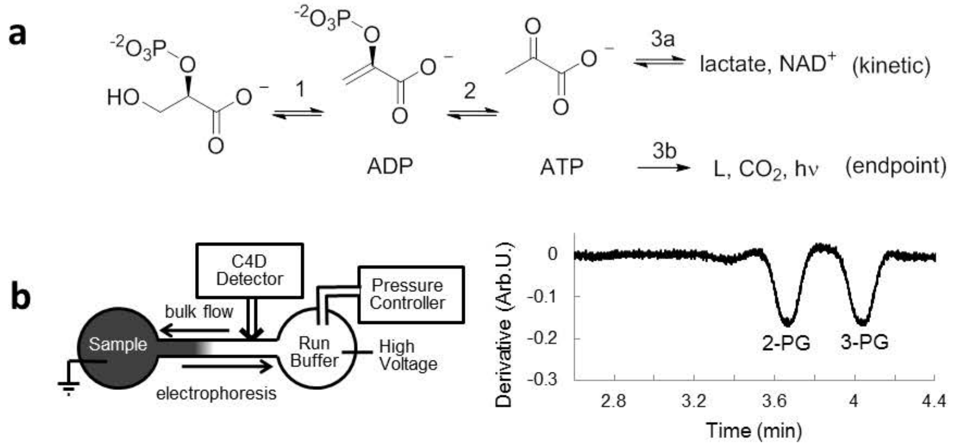

Supplementary Figure 2. *Assay methods used to detect 2- and 3-phosphoglycerate* (**a**) Coupling enzymes used in kinetic NADH absorbance and endpoint bioluminescence assays. Coupling enzymes and substrates are 1, enolase, 2-PG; 2, pyruvate kinase, phosphoenolpyruvic acid (PEP), ADP; 3a, lactate dehydrogenase, pyruvic acid, NADH; and 3b, luciferase, ATP, luciferin (LH2). The products of 3a are NAD^+; 3b, oxyluciferin (L), CO_2 and light (hν). (**b**) Schematic of gradient elution moving boundary capillary electrophoresis (GEMBE) device use in the direct detection of 2-PG / 3-PG (left) and 1st derivative of current detected for phosphoglycerate isomers.

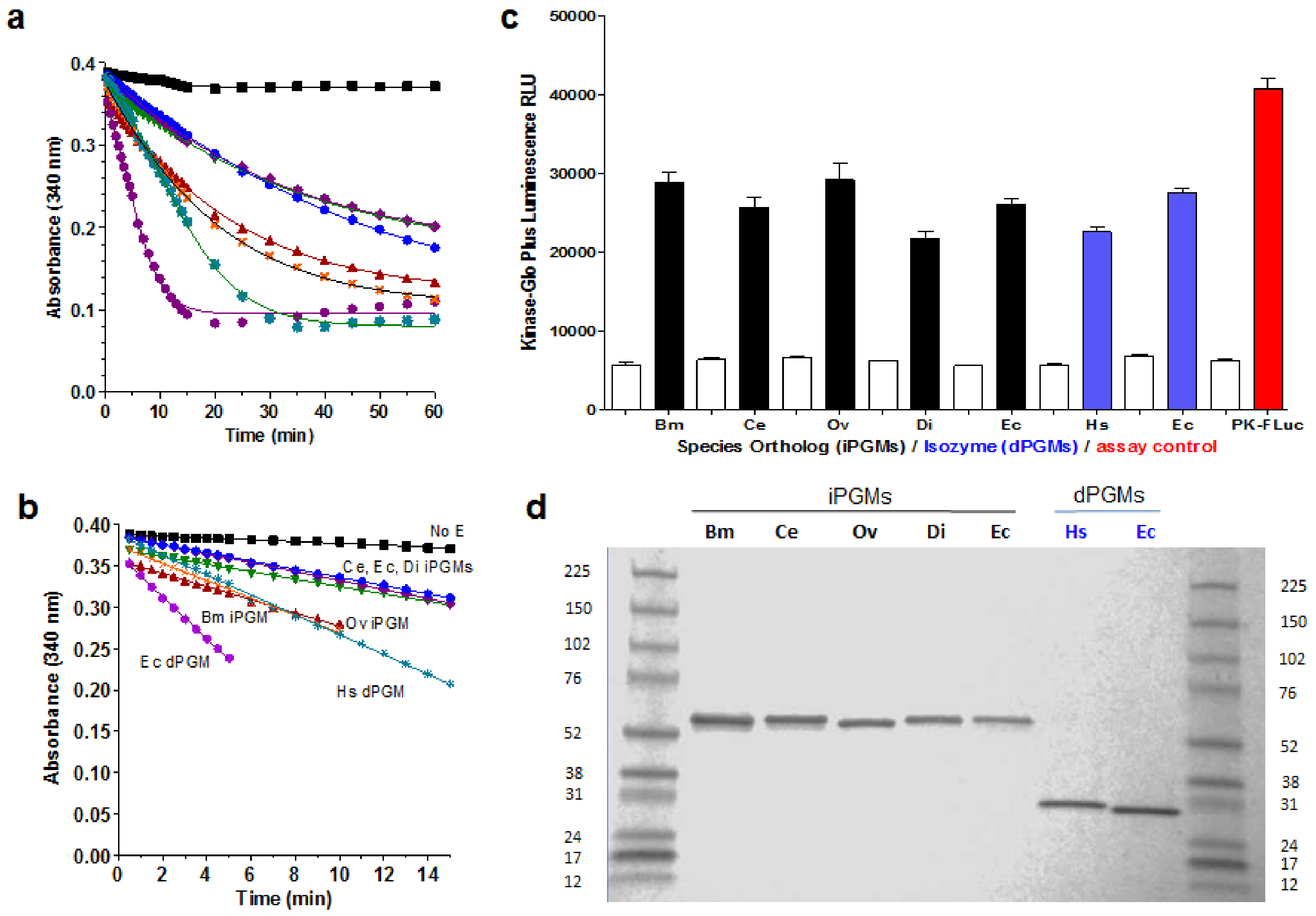

Supplementary Figure 3. *Phosphoglycerate mutase coupled-enzyme assays* (**a**) Reaction time course (1 hour) as measured by a continuous NADH-dependent absorbance assay. (**b**) Linear phase of assays occurs over initial 15 minutes. (**c**) Representative calibration of bioluminescent end-point HTS assays for seven PGM orthologs and PK-FLuc control. Enzyme concentrations (*B. malayi* iPGM, 5 nM; *C. elegans* iPGM, 5 nM; *D. immitis* iPGM, 10 nM; *E. coli* iPGM, 10 nM; *O. volvulus* iPGM, 20 nM; *H. sapiens* dPGM, 5 nM; *E. coli* dPGM, 4 nM; PK, 0.15 units) were adjusted to give approximately equivalent RLU after a 5 min assay time. Open bars are ‘no PGM’ controls for background measurements. Error bars are s.d. from 24 replicates. (**d**) 4-12% SDS-PAGE of enzymes used in assays, 1 μg of each loaded. Abbreviations: *B. malayi*, Bm; *C. elegans*, Ce; *D. immitis*, Di; *E. coli*, Ec; *O. volvulus* Ov; *H. sapiens*, Hs.

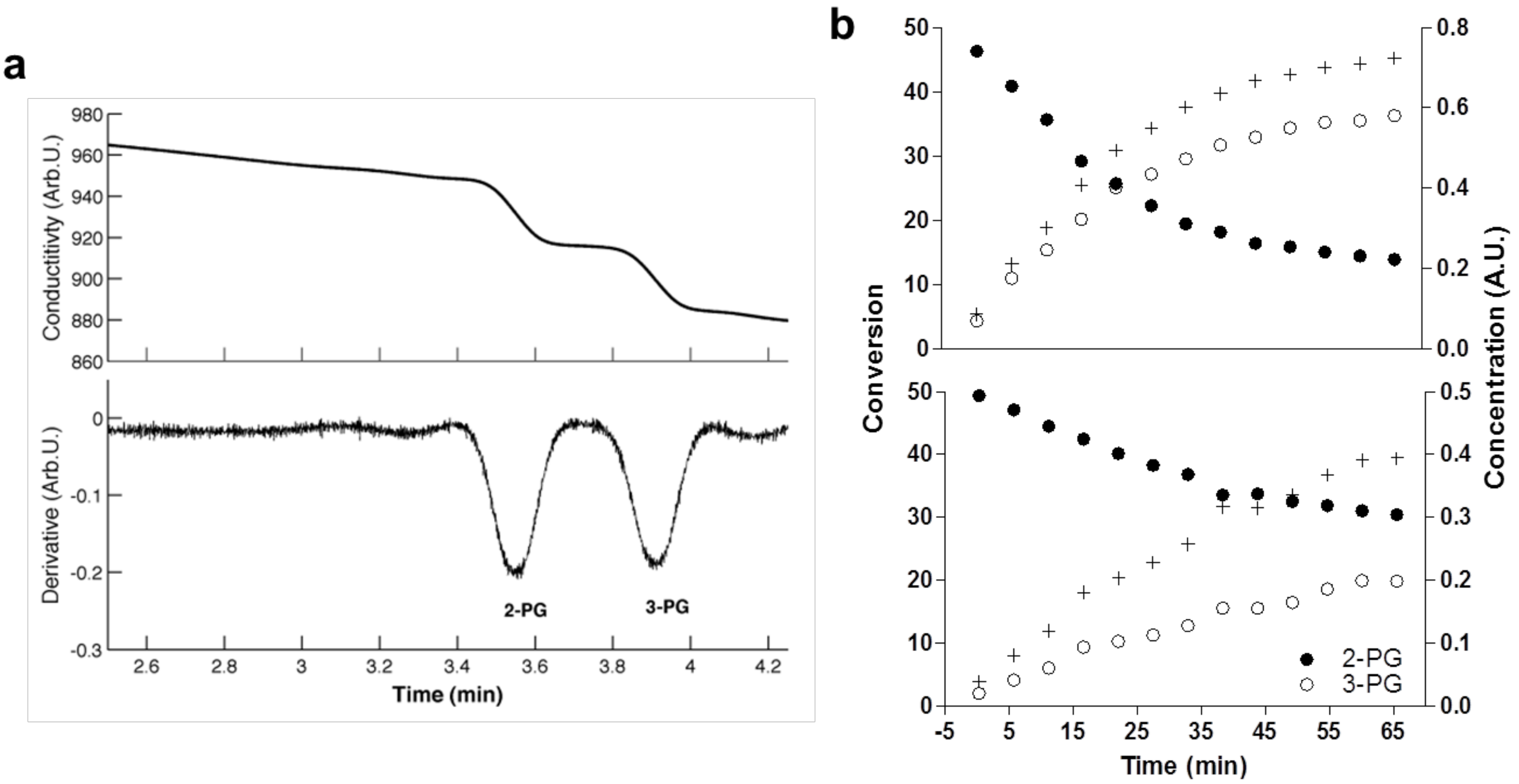

Supplementary Figure 4. *Separation of phosphogycerates with GEMBE*. (**a**) Current and 1st derivative plots for the elution of 2- and 3-PG. (**b**) Time course for the conversion of 3-PG to 2-PG measured with GEMBE, for the *C. elegans* iPGM (top) and the *B. malayi* iPGM (bottom). Right axis concentration of (●) 2-PG, (○) 3-PG (arbitrary units); left axis % conversion of 2-PG to 3-PG.

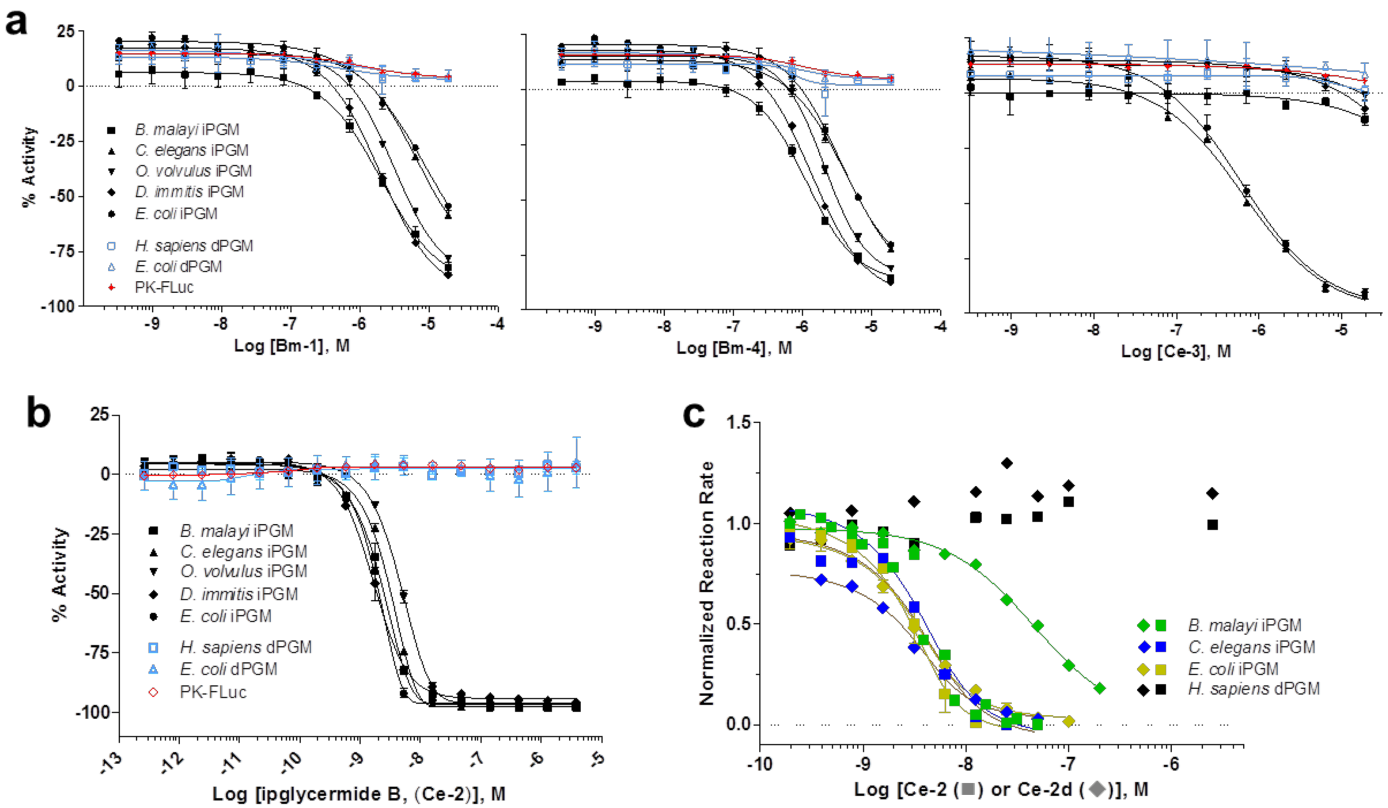

Supplementary Figure 5. *Representative concentration response curves (CRCs) for iPGM inhibitory activity of **Bm-** and **Ce-** cyclic peptides and GEMBE analysis of **Ce-2** and **Ce-2d**.* (**a**) CRCs for **Bm-1**, **Bm-4** and **Ce-3**. (**b**) CRCs for **Ce-2.** Significant deviation from a hyperbolic response required a 5 parameter Hill equation to fit the iPGM data in this plot. Data from the iPGM orthologs and dPGM isozymes determined from the enzyme-coupled bioluminescent assay. The iPGM concentrations for *C. elegans, B. malayi* was 5 nM, for *D. immitis, E. coli* 10 nM , *O. volvulus* 20 nM, and *E. coli*, *H. sapiens* dPGM, 5 nM. Plots are representative from experimental replicates listed in Supplementary Table 3, error bars are defined as described in Supplementary Table 3. (**c**) Concentration-response curves for **Ce-2** (square symbol) and **Ce-2d** (diamond symbol) from the GEMBE assay. Green: *B. malayi* iPGM, blue: *C. elegans* iPGM, amber: *E. coli* iPGM, black: *H. sapiens* dPGM. Treated with inhibitor **Ce-2** (squares) or **Ce-2d** (diamonds). pIC_{50} values from experimental replicates (N) and indicated concentration of enzyme: **Ce-2** (3.4 nM *B. malayi* iPGM) 8.37±0.04 (2); (4.6 nM *C. elegans* iPGM) 8.36 ±0.03 (1); (25 nM *E. coli* iPGM) 8.49 ±0.03 (3); (2.1 nM *H. sapiens* dPGM) no apparent activity **Ce-2d** (3.4 nM *B. malayi* iPGM) 7.21 ±0.19 (1); (4.6 nM *C. elegans* iPGM) 8.49 ±0.02 (1); (25 nM *E. coli* iPGM) 8.50 ±0.02 (3); (2.1 nM *H. sapiens* dPGM) no apparent activity. Data represent mean ± s.d. values of the log normal distributed IC50s determined for the given peptide for experiments with ≥4 replicates (N≥2); otherwise error bar is determined from the nonlinear fit of the standard Hill equation to the aggregated data from the replicates.

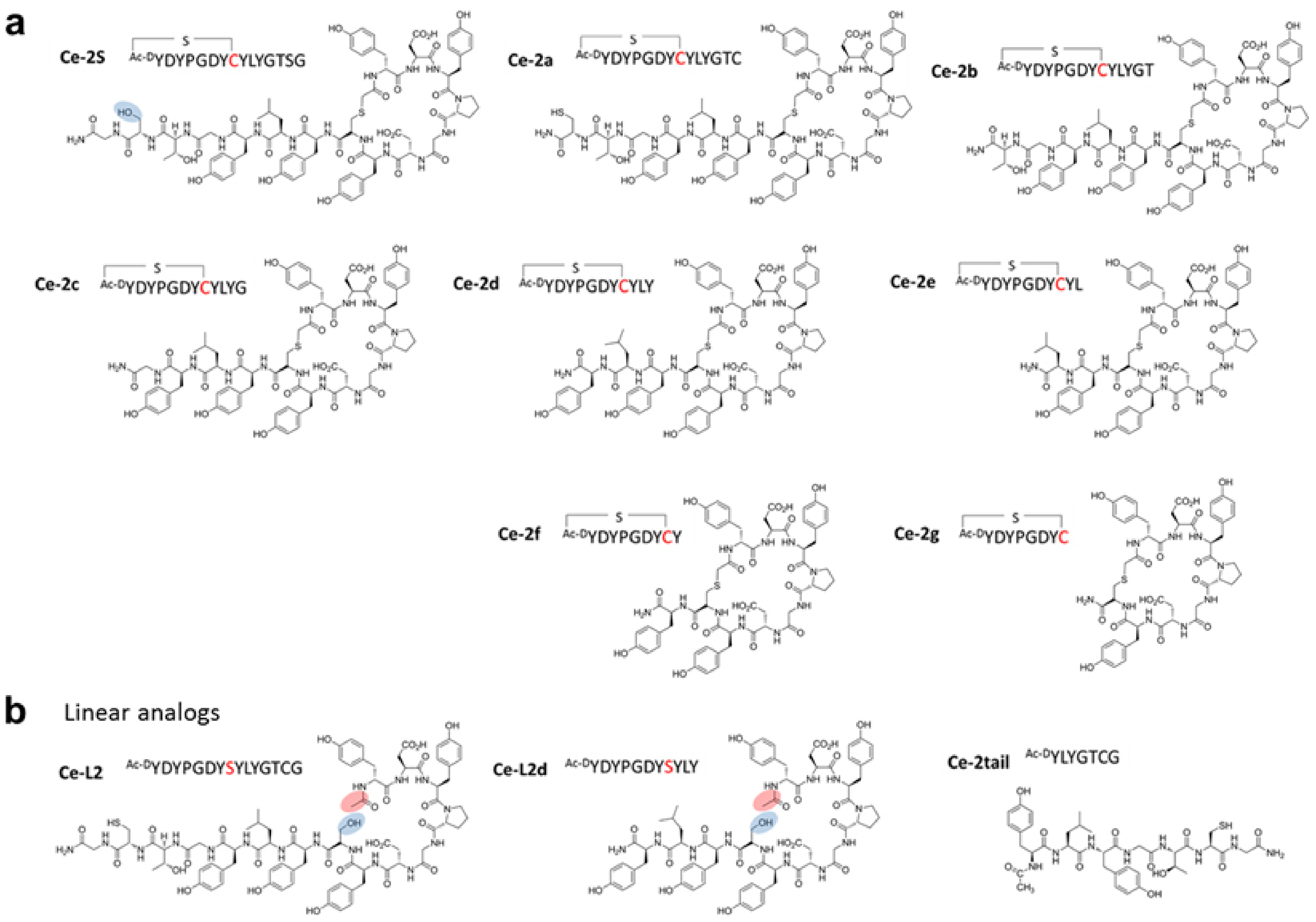

Supplementary Figure 6. *Summary of macrocyclic peptide analog structures.* Hydroxyl groups are shaded in blue and methyl groups in salmon to indicate substitutions.

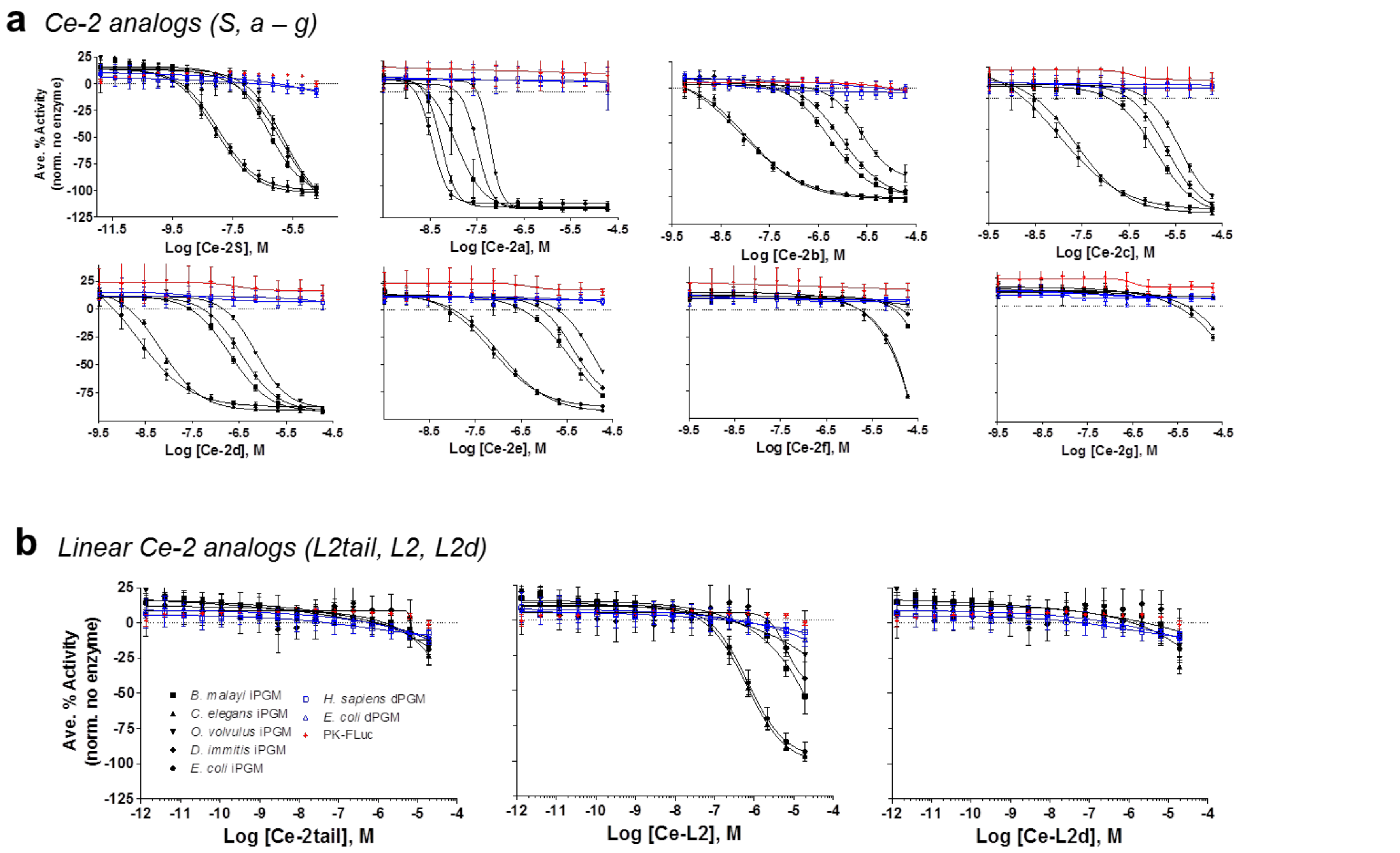

Supplementary Figure 7. *PGM panel activity for macrocyclic peptides analogs of **Ce-2**.* (**a**) **Ce-2S** is a Cys14Ser substitution. **Ce-2a**—**g** are C-terminal amino acid truncation analogs and (**b**) linear peptides (**Ce-L2**, **Ce-L2d** and **Ce-2*tail***). *B. malayi* iPGM (■), *C. elegans* iPGM (▲), *O. volvulus* iPGM (▼), *D. immitis* iPGM (◆), *E. coli* iPGM (●), *H. sapiens* dPGM (□), *E. coli* dPGM (△) and PK-FLuc (●). Plots are representative from experimental replicates listed in Supplementary Table 3, error bars are defined as described in Supplementary Table 3.

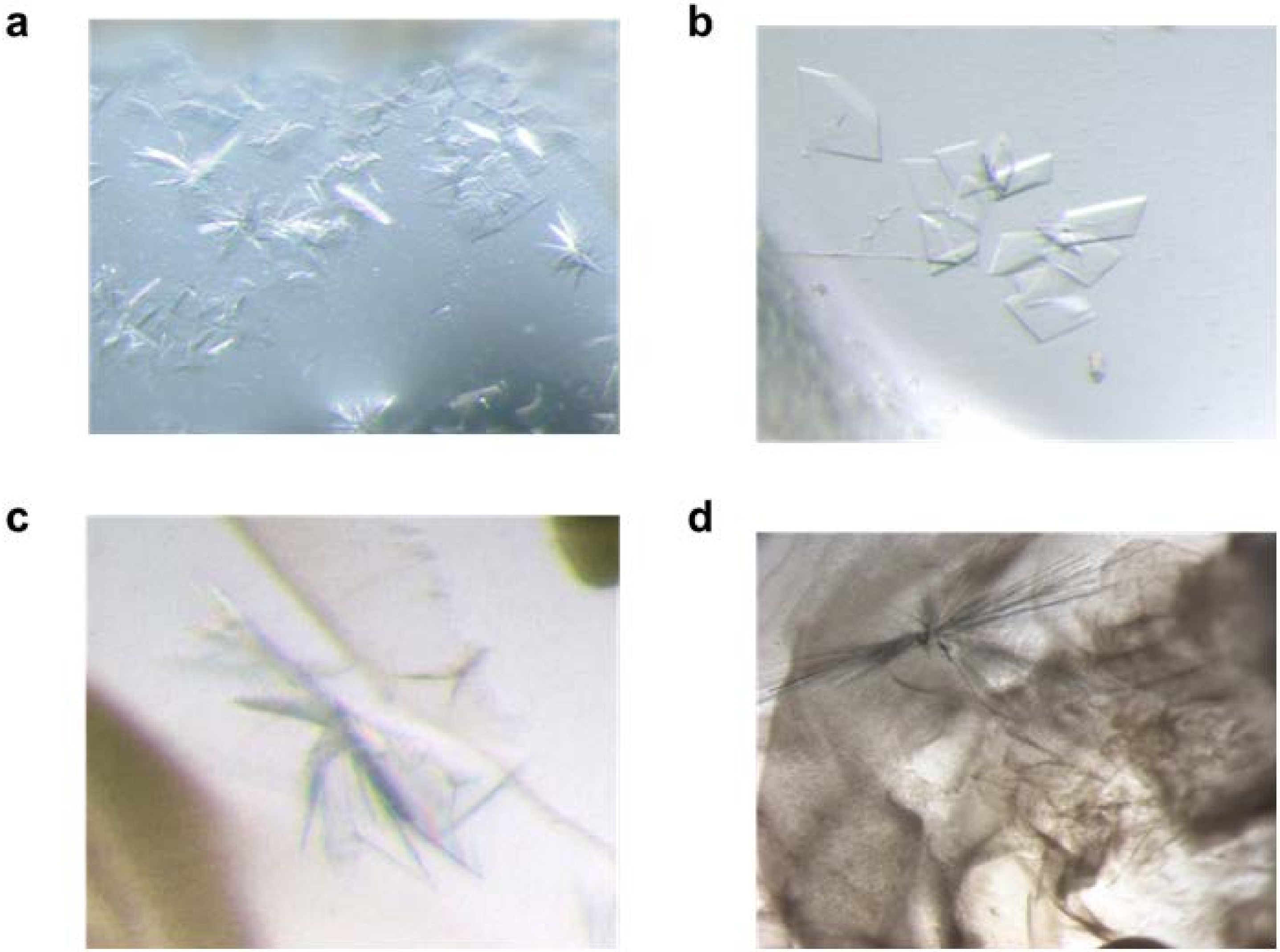

Supplementary Figure 8. *Crystals of apo C. elegans iPGM and in a complex with **Ce-2d**.* (**a**) Initial samples of monoclinic *P* iPGM (iPGM-m) obtained from Wizard 3-4 D11 and (**b**) crystals observed from the Hampton Additive HT screen D7. (**c**) Crystals of orthorhombic *P* iPGM (iPGM-o) obtained from Index HT F7. (**d**) Crystals of a co-complex with **Ce-2d**.

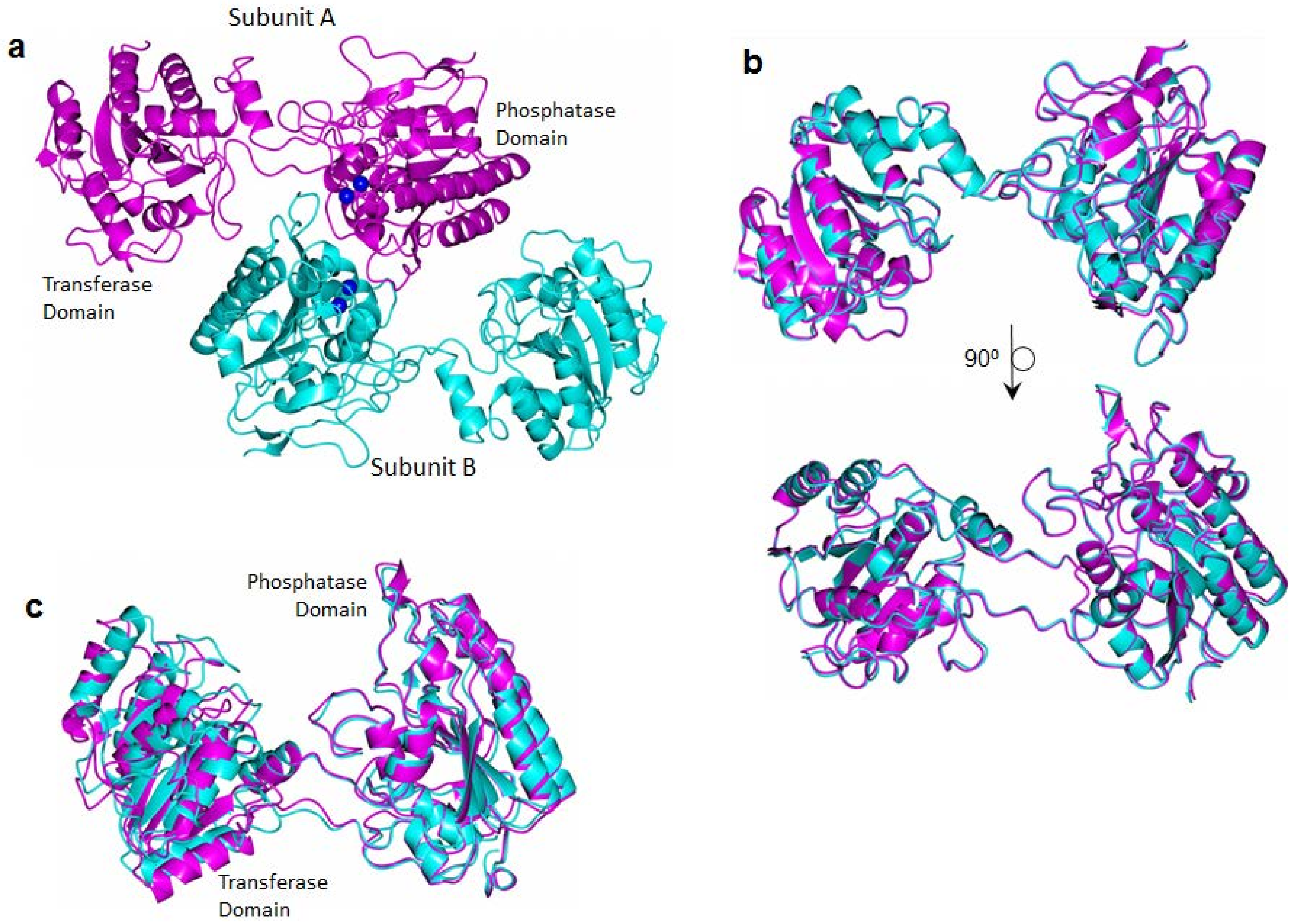

Supplementary Figure 9. *Crystallographic asymmetric unit cell for C. elegans iPGM-m.* (**a**) Asymmetric unit of *C. elegans* iPGM-m showing subunit A (magenta) and B (cyan). The Mn^{2+} and Zn^{2+} ions are represented as blue spheres. (**b**) Comparison of the NCS dimer subunits of *C. elegans* apo iPGM-m showing subunit A (magenta) and B (cyan) superimposed. *Bottom*, same as figure *top* but rotated 90° in the horizontal direction. (**c**) Superposition of *C. elegans* iPGM-m (magenta) and *C. elegans* iPGM-o (cyan).

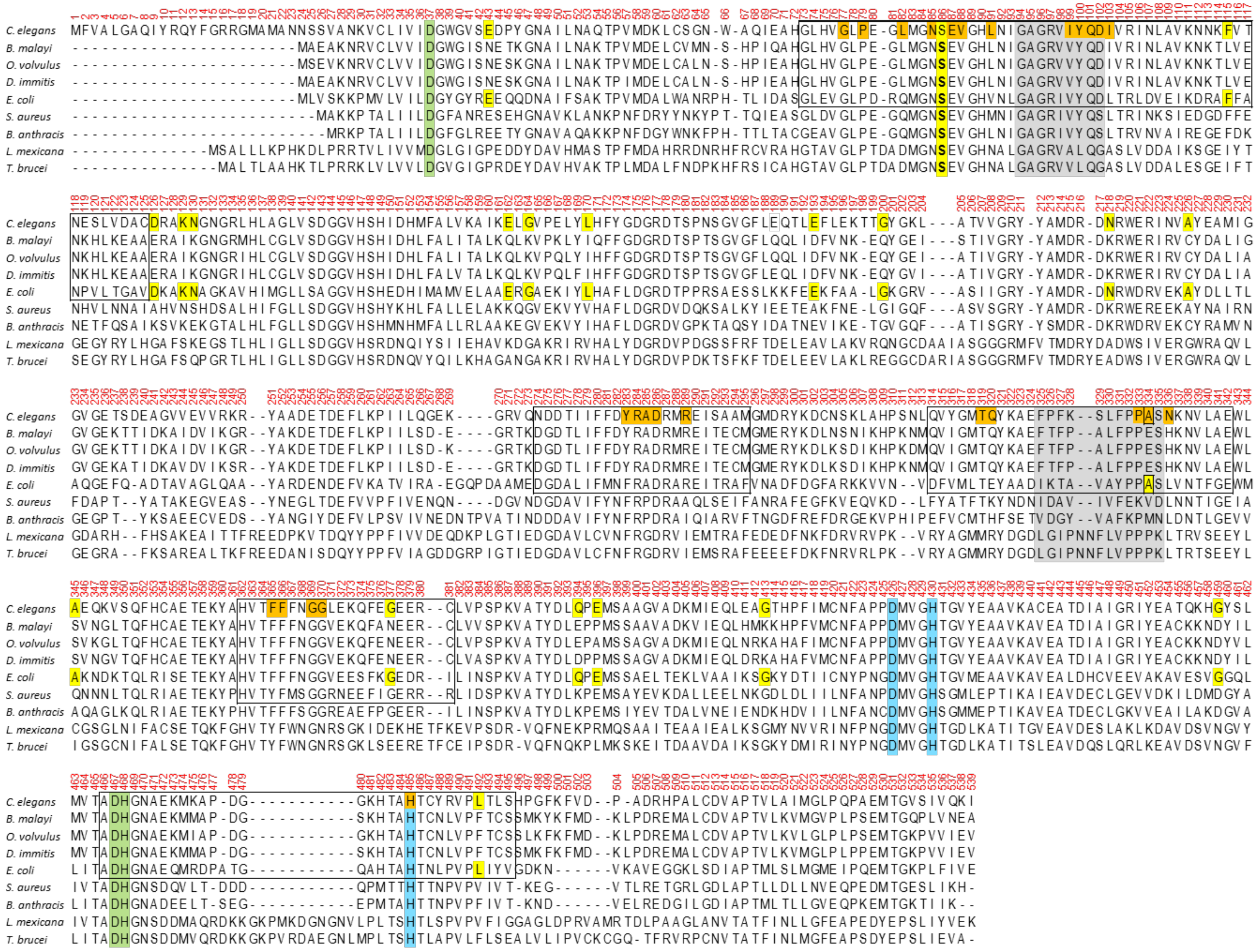

Supplementary Figure 10. Amino acid sequence alignment of various iPGMs. iPGMs residues within 5Å of **Ce-2d** are colored orange. Residues identical between *C. elegans* and *E. coli* iPGM are colored yellow; grey indicates hinge regions; green and blue are amino acids that ligand metal ions. Boxed regions shown in Figure 5b.

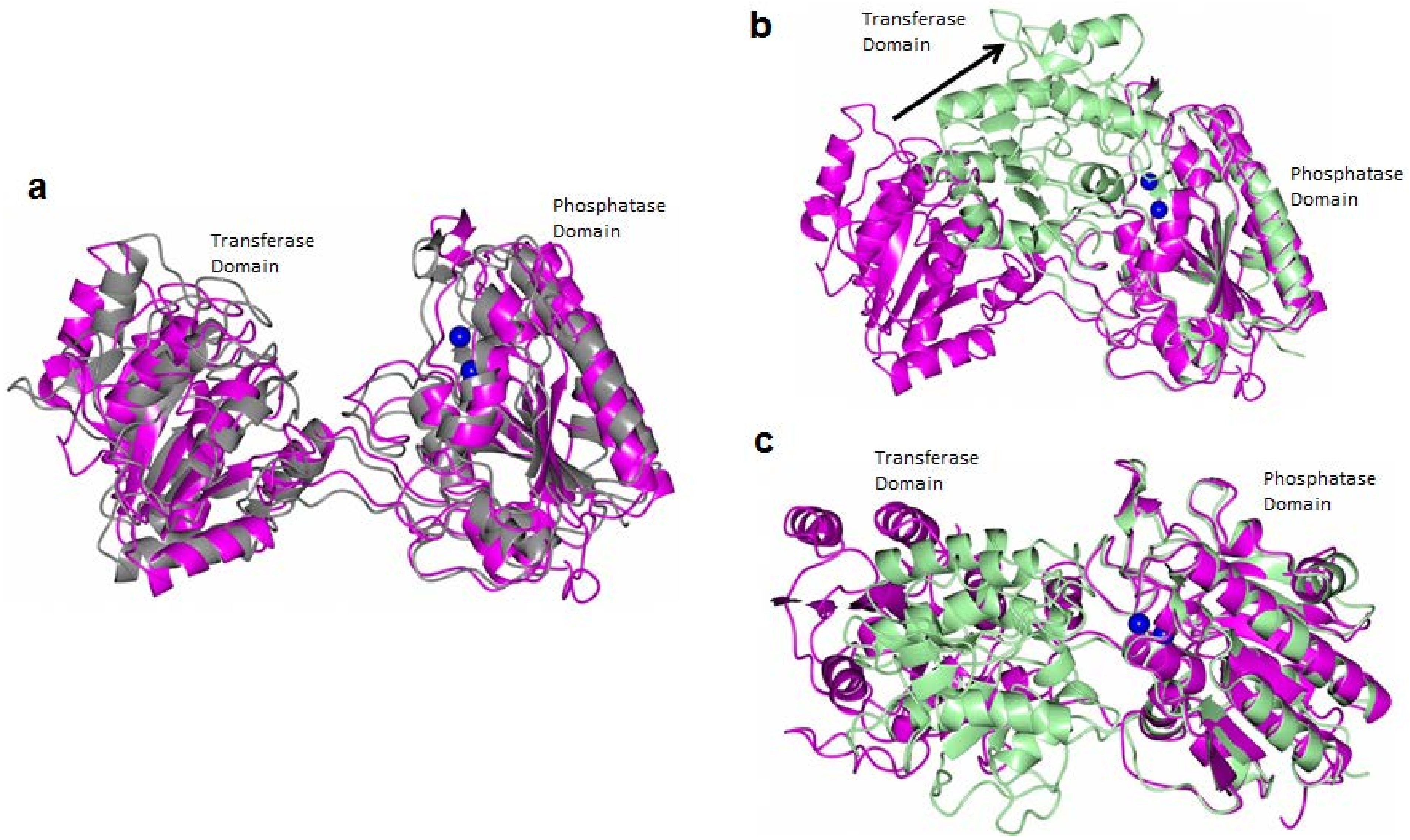

Supplementary Figure 11. *Comparison of C. elegans iPGM to Bacillus anthracis and Bacillus stearothermophilus* (**a**) Superposition of *C. elegans* apo iPGM-m showing subunit A (magenta) and iPGM from *Bacillus anthracis* (gray, PDB: 2IFY) superimposed. Mn^{2+} and Zn^{2+} ions are represented as blue spheres. (**b**) Superposition of *C. elegans* iPGM-m showing subunit A (magenta) and iPGM from *Bacillus stearothermophilus* (green, PDB: 1O98) superimposed. (**c**) Same as above but rotated 90° in the horizontal direction. The arrow indicates the conformational difference in the transferase domain.

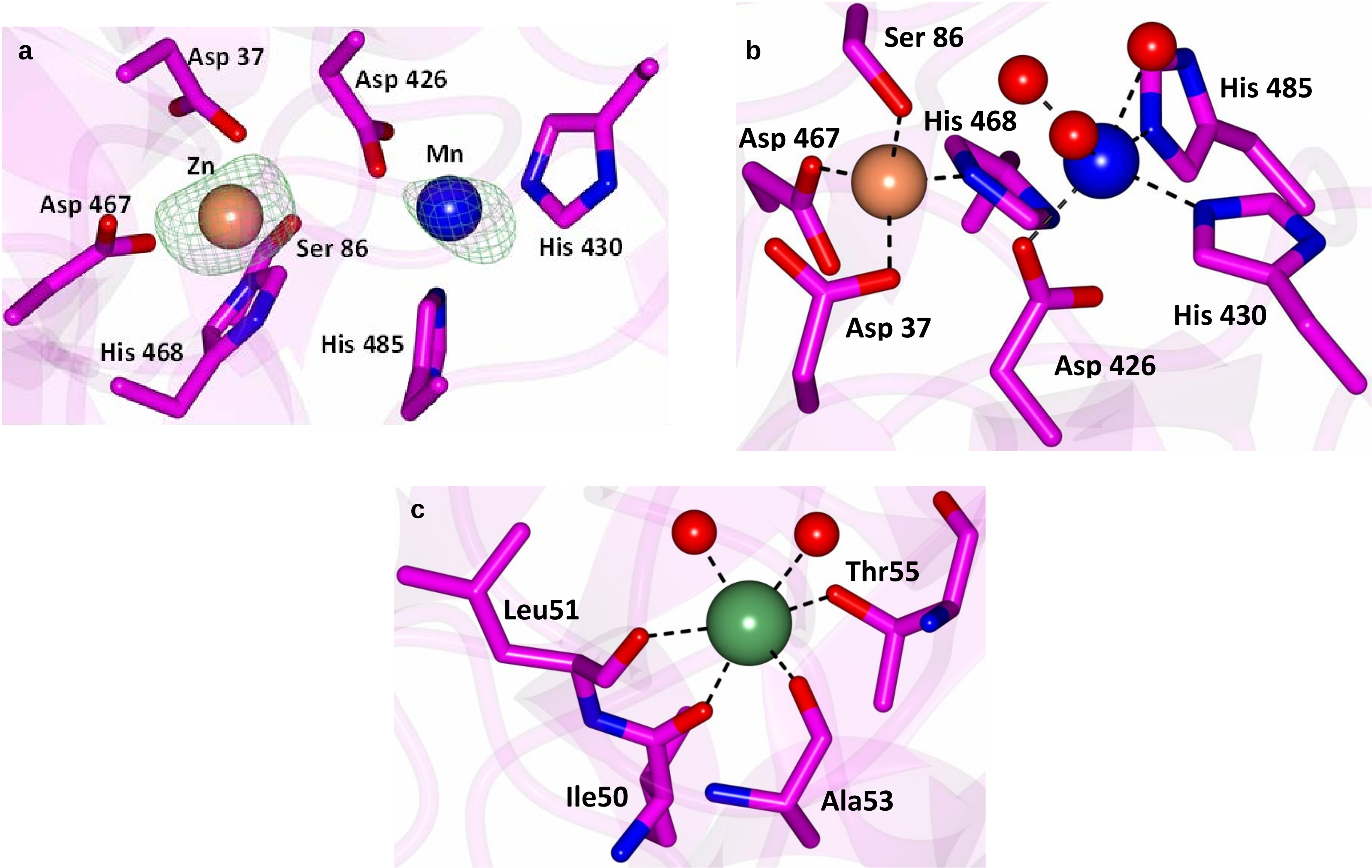

Supplementary Figure 12. *Metal ion binding sites in iPGM* (**a**) Phased anomalous difference map (green mesh) at the metal binding sites of the *C. elegans* iPGM • **Ce-2d** complex contoured at 3σ. (**b**) Metal coordination in the *C. elegans* iPGM • **Ce-2d** complex. Contacts between the protein and Mn^{2+} (blue sphere), Zn^{2+} (tan sphere) ions and water (red spheres). (**c**) Mg^{2+} binding site for iPGM • **Ce-2d**. Mg ion is depicted as a green sphere and water molecules as red spheres.

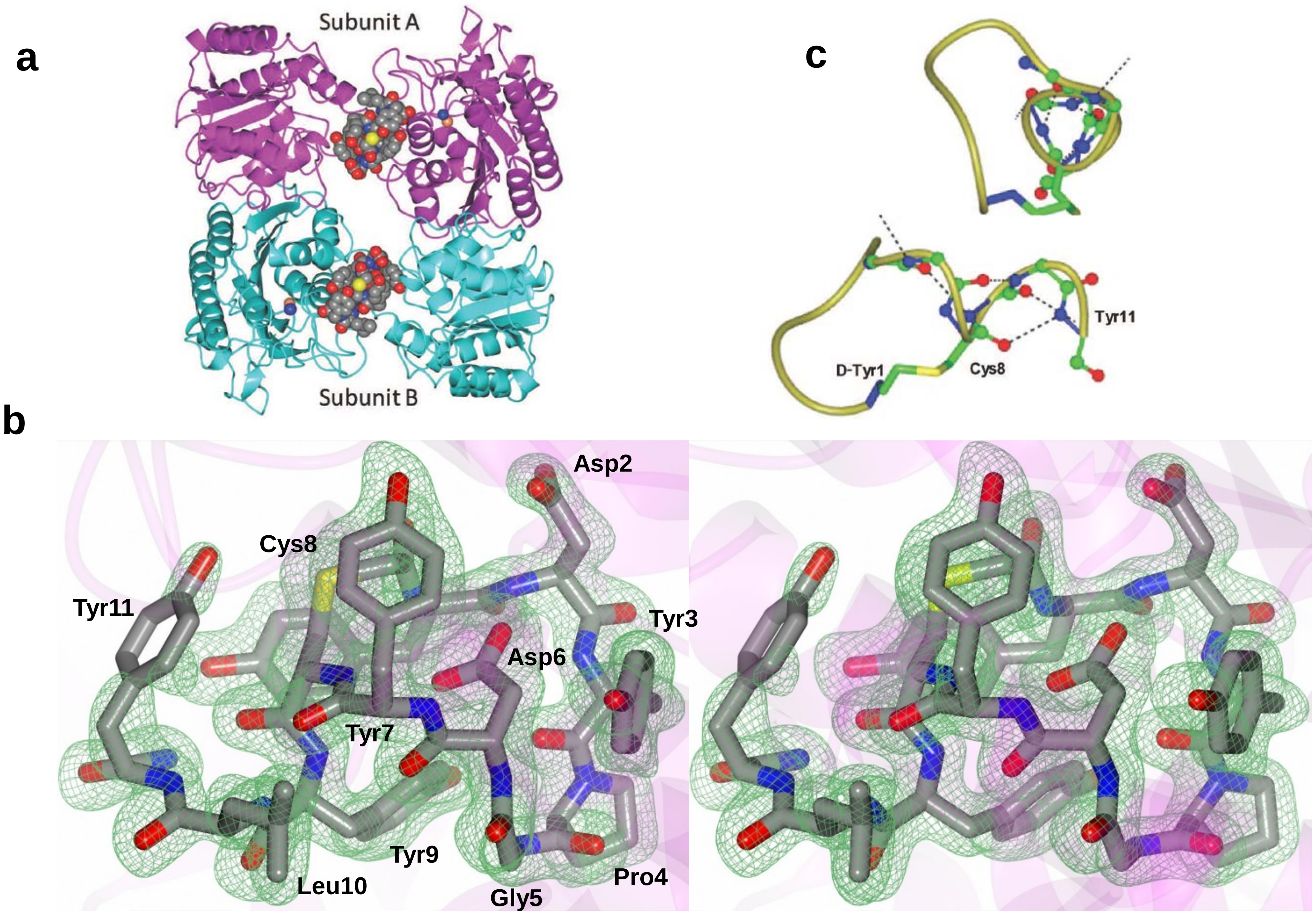

Supplementary Figure 13. *Structure of iPGM•**Ce-2d** complex asymmetric unit.* (**a**) Asymmetric unit of iPGM•**Ce-2d** showing subunit A (magenta) and B (cyan). The Mn^{2+} and Zn^{2+} ions are represented as blue and tan spheres, respectively, and the cyclic peptides bound to each subunit are drawn as gray spheres. (**b**) Wall-eyed stereo image of electron density map (green mesh, Fo-Fc omit) contoured at 3σ for the peptide associated with subunit A (magenta) with numbering for the **Ce-2d** peptide. Residue Tyr11 is capped as an amide. (**c**) α-helical structure of **Ce-2d** C-terminus. Gold is backbone trace with side chains involved in α-helix formation shown.

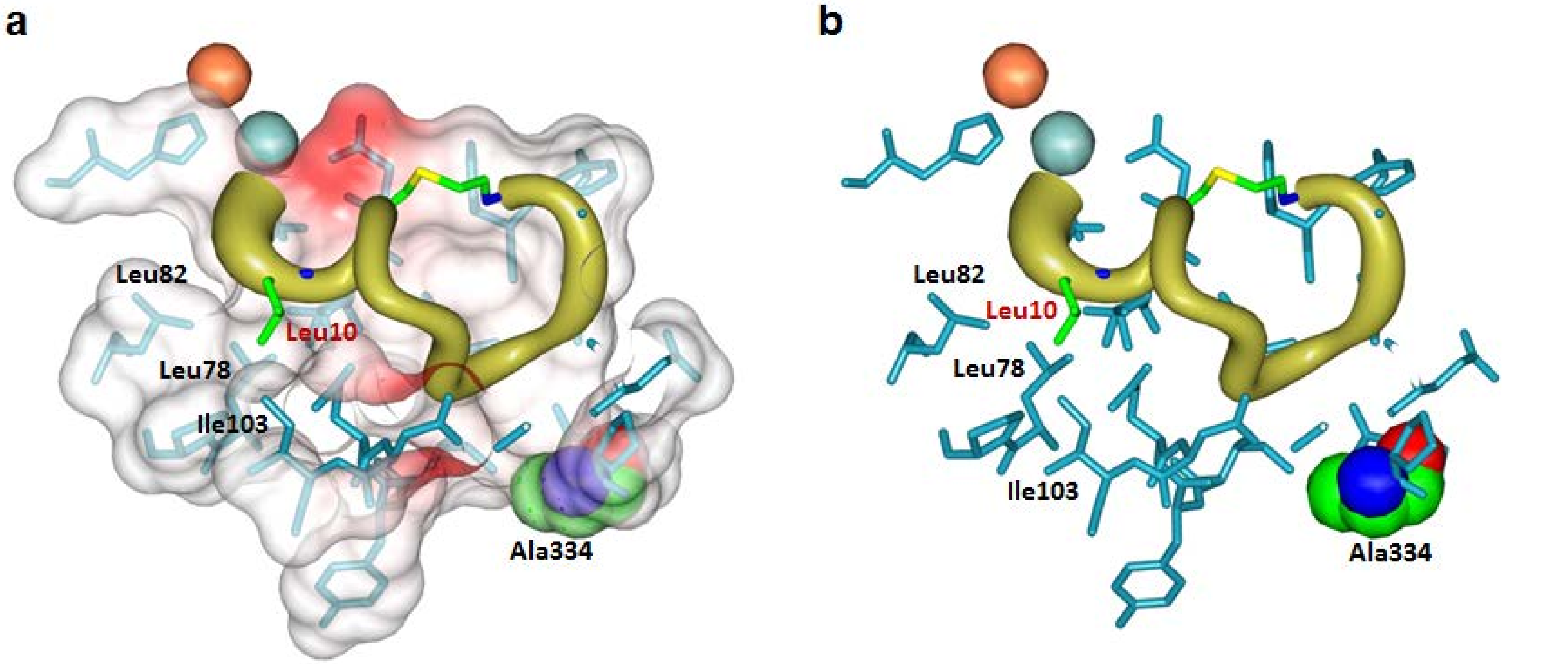

Supplementary Figure 14. *Hydrophobic pocket occupied by Leu10 of* ***Ce-2d****.* (**a**) *C. elegans* iPGM residues (light blue chain under transparent spheres) within 5Å of the **Ce-2d** macrocycle are shown as *B*-factor α-chain (gold) with Leu10 and thioether linkage in green. The iPGM Ala334 residue is shown as a CPK space fill and the Mn^{2+} and Zn^{2+} ions are represented as blue and tan spheres, respectively. (**b**) Same as in (**a**) without transparent spheres for clearer view of iPGM side chains.

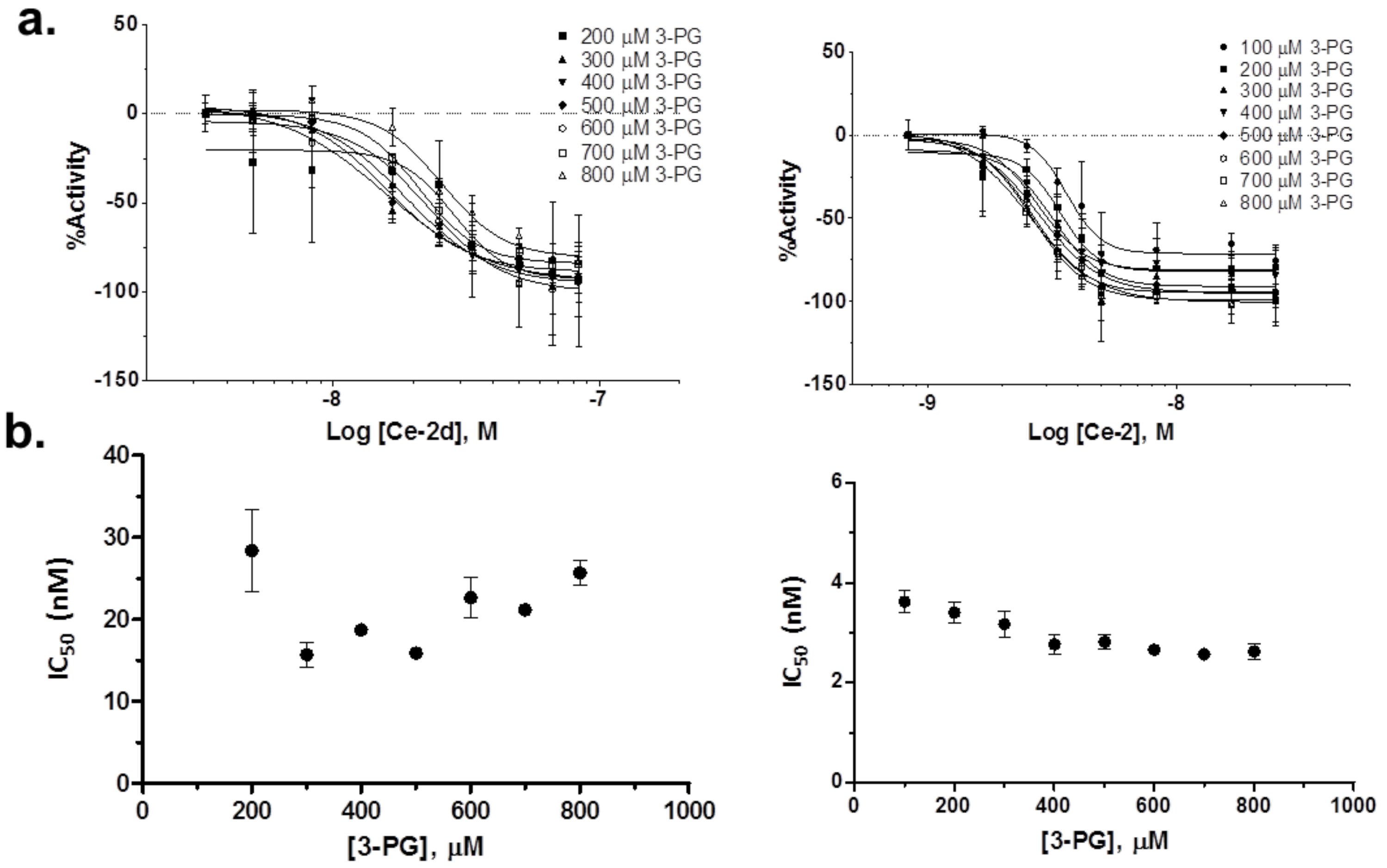

Supplementary Figure 15. *Concentration response curves (**a**) and IC_{50} variation (**b**) for **Ce-2** (right) and **Ce-2d** (left) tested across varying concentrations of 3-PG substrate.* 15 min post substrate addition as measured by a continuous NADH-dependent absorbance assay. The *C. elegans* iPGM concentration used was ~5 and ~15 nM for **Ce-2** and **Ce-2d** respectively. Absorbance values were normalized to no enzyme control and plotted in GraphPad Prism using a 4-paramerter logistic fit for the purpose of estimating IC_{50} values. Error bars represent standard deviation of four replicates. K_M of 3-PG = 200 μM as calculated in this study.

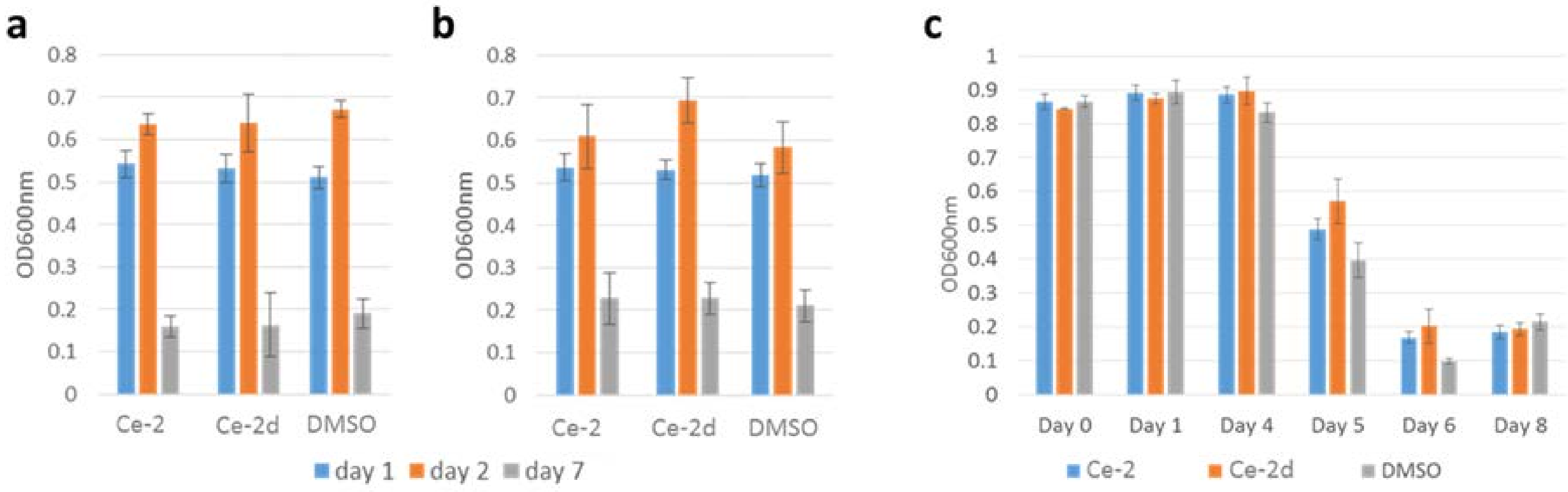

Supplementary Figure 16. *Evaluation of* ***Ce-2*** *and* ***Ce-2d*** *for in vivo activity in C. elegans culture assay.* L1 stage *C. elegans* exposure to 5 μM (**a**) 10 μM (**b**) **Ce-2** or **Ce-2d** for 1 (blue bars), 2 (orange bars) and 7 (grey bars) days. Microscopic examination showed slightly decreased number of F1 progeny in the presence of peptide. (**c**) L4 stage *C. elegans* exposure to 50 μM **Ce-2** (blue bars), 50 μM **Ce-2d** (orange bars), DMSO (grey bars). Data represent the mean ± s.d. of triplicate samples.

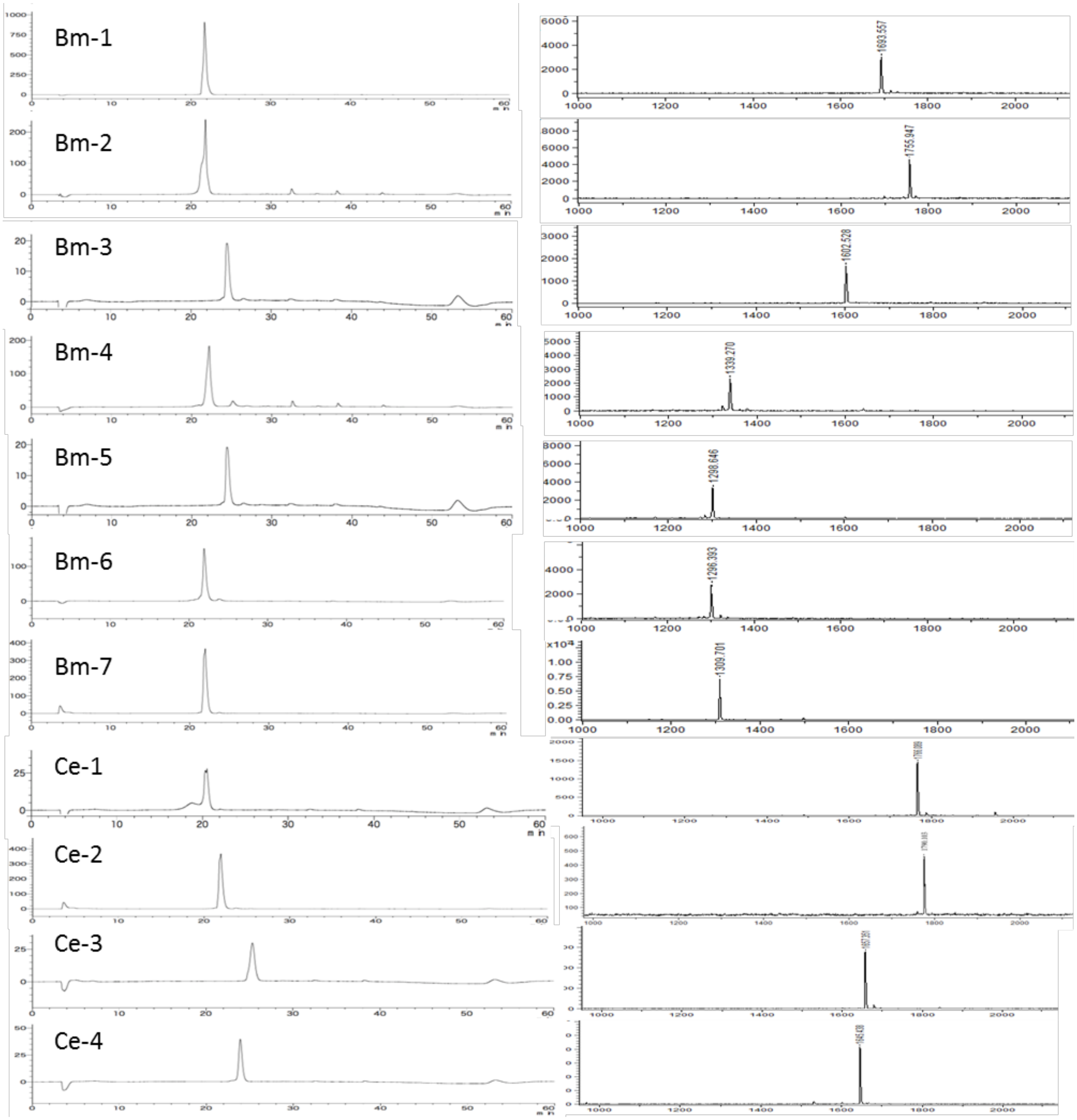

Supplementary Figure 17a. *HPLC profiles (left panel) and corresponding MALDI-TOF mass spectra (right panel) of chemical synthesized peptides described in **Table 1**.* The peptides were dissolved in 30% acetonitrile and loaded onto the column. The peptides were eluted with linear gradient of solution A (0.1% TFA aqueous)and solution B (0.1% TFA acetonitrile) and were detected by UV absorbance at 215nm. Exact masses were measured by MALDI-TOF (Microflex or Autoflex). See **Supplementary Table 4** for peak values.

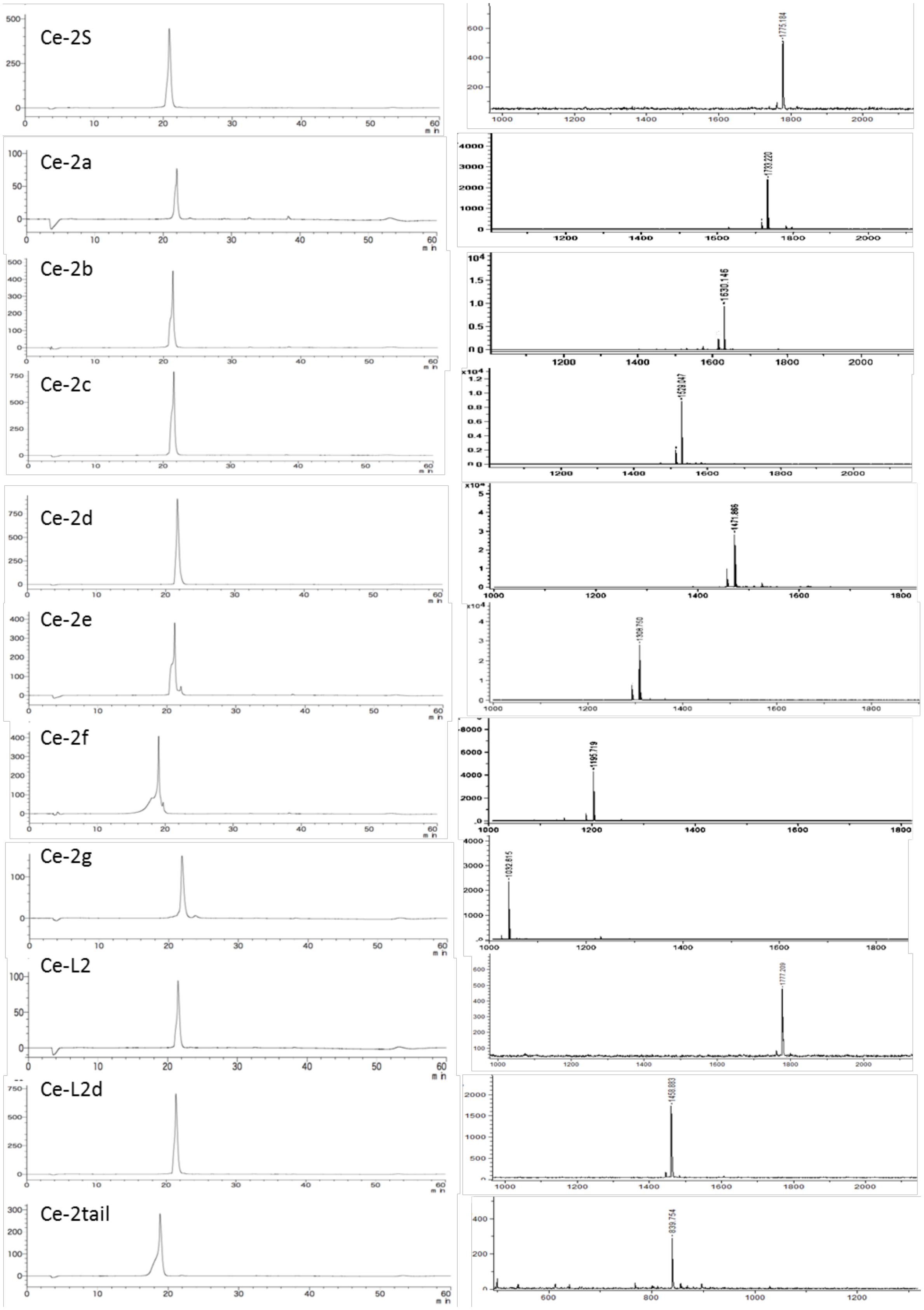

Supplementary Figure 17b. *HPLC profiles (left panel) and corresponding MALDI-TOF mass spectra (right panel) of chemical synthesized peptides described in **Table 2**.* The peptides were dissolved in 30% acetonitrile and loaded onto the column. The peptides were eluted with linear gradient of solution A (0.1% TFA aqueous)and solution B (0.1% TFA acetonitrile) and were detected by UV absorbance at 215nm. Exact masses were measured by MALDI-TOF (Microflex or Autoflex). See **Supplementary Table 4** for peak values.

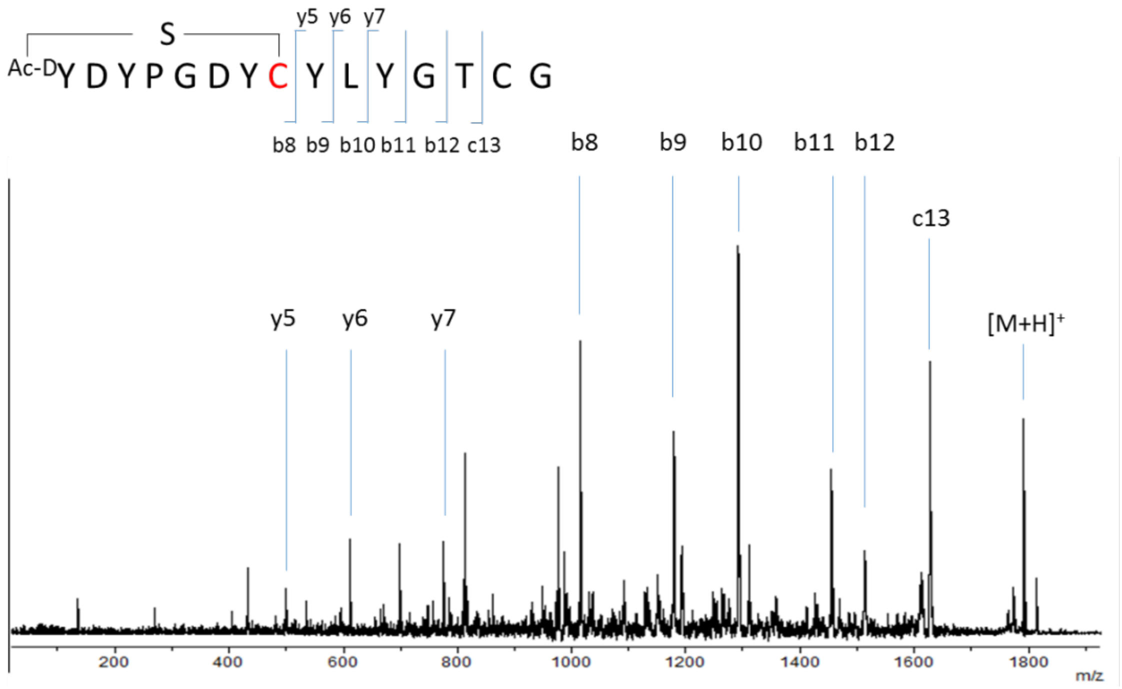

Supplementary Figure 18. *MSMS spectrum and fragment analysis of* ***Ce-2*** *by MALDI-TOF/TOF.* b-type, y-type and c-type fragment ions were indicated by number. The parent ion $[M+H]^+$ of **Ce-2** is 1790.66.

Supplementary Table 1. Assay protocol table

Phosphoglycerate mutase ortholog and isozyme 1536-well plate assay protocol				
**Step**	**Parameter**	**Value**	**Target**	**Description**
1a	Reagent	4 µL	*B. malayi* iPGM	No Enzyme control and Enzyme solutions (5 nM *B. malayi* iPGM, 5 nM *C. elegans* iPGM, 5 nM *H. sapiens* dPGM, 20 nM *O. volvulus* iPGM, 10 nM *D. immitis* iPGM, 10 nM *E. coli* iPGM & 4 nM *E. coli* dPGM final concentrations); white/solid bottom high base plate (Greiner)
1b	Reagent	4 µL	*C. elegans* iPGM	
1c	Reagent	4 µL	*H. sapiens* dPGM	
1d	Reagent	4 µL	*O. volvulus* iPGM	
1e	Reagent	4 µL	*D. immitis* iPGM	
1f	Reagent	4 µL	*E. coli* iPGM	
1g	Reagent	4 µL	*E. coli* dPGM	
1h	Reagent	4 µL	PK-FLuc ctrl	0.15 U PK final concentrations; white/solid bottom high base plate (Greiner)
2	Cyclic Peptides	23 nL		Cyclic Peptides profiled acorss iPGM orthologs and dPGM isozymes [5 mM – 84.7 nM: 19.1 µM – 324.6 pM; 11-pt 1:3 Titration Series] or vehicle (DMSO) control; Peptides transfer by Pintool; Control = No Enzyme
3	Incubation	20-30 min		Peptide interaction with enzyme
4	Reagent	2 µL		3-phosphoglycerate (3PG) and PEP; PEP only for PK control
5	Incubation	10 min		Ambient temperature; dark
6	Reagent	3 µL		Kinase-Glo Plus reagent
7	Incubation	20-30 min		Ambient temperature; dark
8	Measurement	ViewLux		Luminescence mode, 1 sec expos; gain=med.; speed=slow; binning=2X

**Step**	**Notes**
1	Assay buffer: 30mM Tris-HCl , pH 8.0, 5 mM $MgSO_4$, 20 mM KCl, 0.12% BSA + 6 – 30 nM PGM enzyme or 0.0375 units/µl pyruvate kinase
	5X Assay Buffer: 150 mM Tris-HCl pH 8, 25 mM $MgSO_4$, 100 mM KCl, 0.6% BSA
4	Substrate buffer for PGM enzymes: 30mM Tris-HCl , pH 8.0, 5 mM $MgSO_4$, 20 mM KCl, 9 mM ADP, and 0.15 units/µl each of enolase and pyruvate kinase + 1.2 mM 3PG
	Substrate buffer for PK enzyme: 30mM Tris-HCl , pH 8.0, 5 mM $MgSO_4$, 20 mM KCl, 9 mM ADP + 1.2 mM PEP
	5X Assay Buffer: 150 mM Tris-HCl pH 8, 25 mM $MgSO_4$, 100 mM KCl
	Final PGM assay buffer concentrations: 30mM Tris-HCl , pH 8.0, 5 mM $MgSO_4$, 20 mM KCl, 3 mM ADP, and 0.3 units each of enolase and pyruvate kinase + 4 – 20 nM PGM enzyme and 0.4 mM 3PG (or 0.15 units PK + 0.4 mM PEP)

Supplementary Table 2. Assay Summary Statistics

PGM	Output Signal (RLU)	S:B	CV	Z' Factor	Control condition
B. malayi iPGM	22000 ± 6500	4.4 ± 1.8	3.4 ± 1.0	0.81 ± 0.06	No Enzyme
C. elegans iPGM	19000 ± 5400	3.6 ± 1.5	4.0 ± 1.3	0.76 ± 0.11	No Enzyme
O. volvulus iPGM	25000 ± 6300	4.7 ± 2.2	4.7 ± 2.1	0.79 ± 0.10	No Enzyme
D. immitis iPGM	20000 ± 7000	3.8 ± 2.2	4.2 ± 2.4	0.74 ± 0.15	No Enzyme
E. coli iPGM	17000 ± 4100	3.1 ± 1.2	4.0 ± 2.1	0.72 ± 0.16	No Enzyme
H. sapiens dPGM	19000 ± 3200	3.7 ± 1.4	4.0 ± 0.9	0.75 ± 0.10	No Enzyme
E. coli dPGM	23000 ± 3900	3.8 ± 1.5	3.7 ± 1.3	0.77 ± 0.11	No Enzyme
PK-FLuc	29000 ± 5300	5.2 ± 2.0	3.3 ± 1.0	0.77 ± 0.18	No Enzyme

Supplementary Table 3. Activity (pIC_{50} and Max Response) of macrocyclic peptides on PGM Panel

Compound ID	iPGM Ortholog															PGM Isozyme						Control		
	B. malayi iPGM			*C. elegans* iPGM			*O. volvulus* iPGM			*D. immitis* iPGM			*E. coli* iPGM			*H. sapiens* dPGM			*E. coli* dPGM			*PK-Fluc*		
	Max Inhibition	pIC50, M	N	Max Inhibition	pIC50, M	N	Max Inhibition	pIC50, M	N	Max Inhibition	pIC50, M	N	Max Inhibition	pIC50, M	N	Max Inhibition	pIC50, M	N	Max Inhibition	pIC50, M	N	Max Inhibition	pIC50, M	N
**Bm-1**	-82.8 ± 0.9	5.89 ± 0.27	2	-60.5 ± 2.8	5.30 ± 0.08	2	-82.1 ± 5.4	5.88 ± 0.53	2	-85.9 ± 0.2	5.99 ± 0.41	2	-60.6 ± 9.0	5.31 ± 0.06	2	3.0 ± 0.2	NA	2	7.8 ± 4.3	NA	2	10.4 ± 9.0	NA	2
**Bm-2**	-0.1 ± 1.3	NA	2	-7.9 ± 6.2	NA	2	-15.2 ± 26.6	NA	2	-10.5 ± 20.0	NA	2	-12.3 ± 10.6	NA	2	-0.1 ± 1.3	NA	2	-0.1 ± 1.3	NA	2	-0.1 ± 1.3	NA	2
**Bm-3**	-86.4 ± 0.6	5.56 ± 0.29	2	-66.1 ± 7.5	5.05 ± 0.14	2	-89.2 ± 2.0	5.62 ± 0.45	2	-89.4 ± 2.8	5.73 ± 0.47	2	-60.0 ± 3.5	5.03 ± 0.04	2	5.1 ± 0.9	NA	2	8.4 ± 1.0	NA	2	7.9 ± 7.9	NA	2
**Bm-4**	-86.3 ± 1.4	6.24 ± 0.41	2	-75.5 ± 4.3	5.61 ± 0.29	2	-85.7 ± 6.5	6.10 ± 0.63	2	-88.1 ± 0.8	6.21 ± 0.44	2	-76.3 ± 8.3	5.56 ± 0.16	2	6.1 ± 2.2	NA	2	7.3 ± 3.1	NA	2	9.6 ± 6.4	NA	2
**Bm-5**	-84.7 ± 1.2	6.16 ± 0.36	2	-73.9 ± 4.8	5.60 ± 0.29	2	-83.1 ± 7.3	5.99 ± 0.69	2	-87.1 ± 3.2	6.10 ± 0.56	2	-74.3 ± 9.5	5.54 ± 0.20	2	5.5 ± 0.8	NA	2	3.8 ± 4.4	NA	2	7.3 ± 9.7	NA	2
**Bm-6**	-86.8 ± 0.6	6.16 ± 0.30	2	-76.4 ± 1.8	5.58 ± 0.25	2	-86.0 ± 3.2	6.08 ± 0.53	2	-90.2 ± 1.2	6.26 ± 0.43	2	-77.4 ± 4.3	5.55 ± 0.07	2	1.4 ± 3.8	NA	2	7.6 ± 1.1	NA	2	5.7 ± 2.8	NA	2
**Bm-7**	-74.0 ± 10.1	5.28 ± 0.39	2	-78.0 ± 2.0	5.13 ± 0.18	2	-78.0 ± 19.9	5.37 ± 0.52	2	-83.1 ± 9.7	5.42 ± 0.46	2	-69.7 ± 12.3	5.12 ± 0.09	2	7.1 ± 2.6	NA	2	9.3 ± 2.2	NA	2	2.7 ± 3.2	NA	2
**Bm-4a**	-88.0 ± 0.4	6.31 ± 0.29	2	-79.0 ± 0.1	5.61 ± 0.23	2	-87.8 ± 4.1	6.14 ± 0.62	2	-90.5 ± 1.5	6.28 ± 0.40	2	-78.3 ± 3.4	5.58 ± 0.19	2	5.8 ± 0.4	NA	2	8.8 ± 0.7	NA	2	10.7 ± 2.2	NA	2
**Bm-4b**	3.1 ± 2.5	NA	2	2.1 ± 5.6	NA	2	-4.1 ± 19.1	NA	2	3.9 ± 10.9	NA	2	13.6 ± 2.3	NA	2	6.1 ± 3.1	NA	2	9.8 ± 0.4	NA	2	9.9 ± 0.4	NA	2
**Bm-4c**	4.2 ± 7.0	NA	2	2.1 ± 10.6	NA	2	8.7 ± 11.6	NA	2	6.5 ± 10.0	NA	2	6.5 ± 8.2	NA	2	8.2 ± 0.2	NA	2	8.1 ± 4.6	NA	2	6.1 ± 10.0	NA	2
**Bm-4d**	11.2	NA	1	22.7	NA	1	16.9	NA	1	0.7	NA	1	-5.7	NA	1	-1.1	NA	1.0	-1.8	NA	1	4.4	NA	1
**Bm-4e**	-5.9 ± 4.1	NA	2	-33.6 ± 33.8	2.50 ± 3.53	2	-14.9 ± 30.9	NA	2	-21.4 ± 37.3	4.67	2	-20.9 ± 4.5	5.2	2	3.2 ± 2.9	NA	2	8.1 ± 3.0	NA	2	11.5 ± 5.2	NA	2
**Ce-1**	-94 ± 3.4	7.92 ± 0.38	2	-95.8 ± 3.5	8.40 ± 0.07	2	-95.4 ± 2.6	7.61 ± 0.65	2	-95.0 ± 6.0	8.04 ± 0.41	2	-96.5 ± 4.2	8.38 ± 0.10	2	4.0 ± 1.1	NA	2	5.0 ± 10.4	NA	2	7.6 ± 17.6	NA	2
**Ce-2**	-98.8 ± 3.9	8.27 ± 0.47	9	-100.2 ± 6.6	8.65 ± 0.55	9	-98.2 ± 3.4	7.72 ± 0.69	9	-101.1 ± 5.5	8.21 ± 0.60	9	-101.7 ± 5.9	8.78 ± 0.43	9	-0.3 ± 6.3	NA	9	-5.9 ± 6.9	NA	9	-1.4 ± 3.6	NA	9
**Ce-2***	-101.7 ± 1.0	9.02 ± 0.04	4	-100.9 ± 2.4	9.60 ± 0.15	4	-101.5 ± 3.4	9.18 ± 0.08	4	-98.9 ± 5.8	9.36 ± 0.11	4	-105.7 ± 15.1	9.26 ± 0.04	4	1.9 ± 2.7	NA	4	2.7 ± 0.7	NA	4	NT	NT	0
**Ce-3**	-13.3 ± 2.0	NA	2	-92.3 ± 0.4	6.33 ± 0.18	2	-22.9 ± 30.8	5.26 ±0.18	2	-30.1 ± 32.2	5.18 ± 0.03	2	-91.4 ± 1.3	6.27 ± 0.10	2	1.6 ± 0.7	NA	2	9.1 ± 0.8	NA	2	9.5 ± 5.5	NA	2
**Ce-4**	-2.9 ± 3.3	NA	2	-57.0 ± 17.1	5.22 ± 0.17	2	-23.0 ± 35.7	5.37 ±0.14	2	-22.1 ± 35.5	5.28± 0.06*	2	-44.3 ± 1.9	5.28 ± 0.02	2	5.6 ± 3.5	NA	2	8.5 ± 3.3	NA	2	4.7 ± 3.7	NA	2
**Ce-2a**	-99.2 ± 5.3	8.07 ± 0.10	3	-100.7 ± 6.9	8.39 ± 0.11	3	-97.7 ± 4.4	7.31 ± 0.09	3	-100.1 ± 5.6	7.82 ± 0.29	3	-99.2 ± 8.5	8.51 ± 0.14	3	-1.6 ± 4.4	NA	3	-0.9 ± 8.3	NA	3	4.8 ± 7.5	NA	3
**Ce-2a***	-100.3 ± 0.8	9.49 ± 0.08	4	-99.6 ± 3.9	9.76 ± 0.05	4	-97.5 ± 2.7	9.68 ± 0.09	4	-92.5 ± 4.0	9.91 ± 0.09	4	-84.1 ± 9.5	9.58 ± 0.02	4	20.8 ± 1.7	NA	4	9.0 ± 2.0	NA	4	NT	NT	0
**Ce-2b**	-91.8 ± 5.4	6.16 ± 0.14	2	-97.9 ± 8.0	7.80 ± 0.20	2	-80.1 ± 2.1	5.61 ± 0.02	2	-90.8 ± 4.9	5.96 ± 0.10	2	-95.3 ± 9.0	7.98 ± 0.13	2	3.0 ± 3.9	NA	2	3.4 ± 8.1	NA	2	7.2 ± 12.8	NA	2
**Ce-2c**	-88.2	5.93± 0.06	1	-92.4	7.64± 0.03	1	-78.4	5.41± 0.05	1	-85.3	5.70± 0.05	1	-89.1	7.98± 0.12	1	9.6	NA	1	9.7	NA	1	13.4	NA	1
**Ce-2d**	-98.9 ± 4.6	7.17 ± 0.42	6	-102.6 ± 6.6	8.56 ± 0.39	6	-97.0 ± 5.4	6.72 ± 0.60	6	-101.7 ± 6.0	7.04 ± 0.44	6	-102.0 ± 6.9	8.71 ± 0.31	6	-1.2 ± 5.6	NA	6	-6.3 ± 9.9	NA	6	0.0 ± 7.1	NA	6
**Ce-2d***	-100.2 ± 1.4	7.29 ± 0.04	4	-100.4 ± 4.0	9.03 ± 0.06	4	-99.1 ± 3.8	7.13 ± 0.05	4	-94.0 ± 8.2	6.20 ± 1.13	4	-99.4 ± 13.3	7.98 ± 0.27	4	7.9 ± 5.2	NA	4	2.3 ± 1.0	NA	4	NT	NT	0
**Ce-2e**	-82.7 ± 6.1	5.53 ± 0.23	2	-97.2 ± 8.2	7.16 ± 0.26	2	-55.5 ± 1.4	4.72 ± 0.26	2	-76.4 ± 7.4	5.32 ± 0.05	2	-94.5 ± 9.0	7.27 ± 0.16	2	5.3 ± 3.5	NA	2	3.8 ± 5.9	NA	2	8.9 ± 11.2	NA	2
**Ce-2f**	-14.8	NA	1	-79.6	4.29 ± 0.13	1	4.3	NA	1	-3.9	NA	1	-79.6	4.59 ± 0.10	1	7.5	NA	1	10.0	NA	1	18.1	NA	1
**Ce-2g**	6.9	NA	1	-18.7	NA	1	7.4	NA	1	7.4	NA	1	-27.0	NA	1	7.4	NA	1	10.2	NA	1	15.8	NA	1
**Ce-L2**	-48.2 ± 18.2	4.89± 0.21	1	-95.4 ± 8.7	5.94 ± 0.36	3	-40.2 ± 27.6	5.18	3	-44 ± 22.6	5.18 ± 0.17	1	-95.1 ± 5.2	5.88 ± 0.39	3	-1.4 ± 6.9	NA	3	-14.1 ± 1.3	NA	3	-3.0 ± 1.2	NA	3
**Ce-L2d**	-7.3 ± 2.0	NA	3	-20.9 ± 9.2	NA	3	-15.7 ± 8.3	NA	3	-5.2 ± 4.9	NA	3	-21.1 ± 2.4	NA	3	-1.1 ± 8.5	NA	3	-13.4 ± 3.2	NA	3	-2.5 ± 2.3	NA	3
**Ce-2tail**	-10.2 ± 3.3	NA	3	-8.1 ± 13.9	NA	3	-11.6 ± 5.3	NA	3	-1.9 ± 10.3	NA	3	-21.1 ± 2.7	NA	3	-0.4 ± 7.4	NA	3	-13.9 ± 3.8	NA	3	-2.9 ± 3.4	NA	3
**Ce-2S**	-97.7 ± 0.6	6.28 ± 0.17	3	-105 ± 4.7	8.08 ± 0.04	3	-97.0 ± 1.6	5.99 ± 0.28	3	-98.5 ± 0.6	6.01 ± 0.28	3	-104.6 ± 2.5	8.00 ± 0.10	3	1.6 ± 7.0	NA	3	-12.1 ± 5.1	NA	3	-1.4 ± 4.4	NA	3
**Ce-2S***	-77.1 ± 7.5	6.39 ± 0.04	4	-98.6 ± 4.9	7.92 ± 0.08	4	-69.8 ± 9.3	6.35 ± 0.08	4	-63.7 ± 11.4	6.24 ± 0.23	4	-68.1 ± 8.9	7.98 ± 0.27	4	30.5 ± 5.2	NA	4	12.4 ± 1.6	NA	4	NT	NT	0

Error was determined as follows: values for samples with N ≥2 experiments (≥4 replicates) represent s.d. For samples tested twice (N=1) the standard error is determined from the nonlinear fit of the standard Hill equation to the aggregated data from the technical replicates (n=2). NA = no appreciable inhibition, NT= not tested. *Represent values obtained for iPGM concentrations of 500pM to better estimate potency of the higher affinity ligands, Ce-2 and Ce-2a; lower affinity ligands, Ce-2d and Ce-S re-tested as well for comparison.

Supplementary Table 4. Purity and mass analysis of chemically synthesized peptide described in **Tables 1 and 2**

Peptide ID	Purity %	Retention time / min	Observed ion	Calculated exact mass	Observed exact mass
**Bm-1**	>95	21.9	$[M+H]^+$	1692.71	1693.56
**Bm-2**	>90	21.8	$[M+H]^+$	1754.87	1755.95
**Bm-3**	>90	24.4	$[M+H]^+$	1601.68	1602.53
**Bm-4**	>90	22.1	$[M+H]^+$	1338.50	1339.27
**Bm-5**	>90	24.2	$[M+H]^+$	1297.47	1298.65
**Bm-6**	>90	21.9	$[M+H]^+$	1295.46	1296.39
**Bm-7**	>95	21.6	$[M+H]^+$	1308.54	1309.70
**Ce-1**	>90	20.3	$[M+H]^+$	1764.65	1766.09
**Ce-2**	>90	21.9	$[M-H]^-$	1790.66	1790.10
**Ce-3**	>85	25.3	$[M+H]^+$	1655.86	1657.35
**Ce-4**	>90	23.9	$[M+H]^+$	1643.82	1645.43
**Ce-2S**	>95	20.9	$[M+H]^+$	1774.68	1775.18
**Ce-2a**	>90	20.8	$[M-H]^-$	1734.62	1733.22
**Ce-2b**	>95	21.4	$[M-H]^-$	1631.61	1630.15
**Ce-2c**	>95	21.5	$[M-H]^-$	1530.56	1529.05
**Ce-2d**	>95	21.7	$[M-H]^-$	1473.54	1471.89
**Ce-2e**	>90	20.4	$[M-H]^-$	1310.48	1308.75
**Ce-2f**	>90	19.0	$[M-H]^-$	1197.40	1195.72
**Ce-2g**	>90	20.9	$[M-H]^-$	1034.33	1032.62
**Ce-L2**	>95	21.5	$[M+H]^+$	1776.70	1777.21
**Ce-L2d**	>95	21.4	$[M+H]^+$	1458.60	1458.88
**Ce-2tail**	>95	18.8	$[M+Na]^+$	816.35	839.75

Exact masses were measured by MALDI-TOF (Microflex or Autoflex). Purities and retention times are determined by analytical HPLC. The peptides were dissolved in 30% acetonitrile and loaded onto the column. The peptides were eluted with a linear gradient of solution A (0.1% TFA aqueous) and solution B (0.1% TFA acetonitrile) and were detected by UV absorbance at 215nm.

Supplementary Table 5. X-ray data collection and refinement statistics

	C. elegans **iPGM-m**	***C. elegans*** **iPGM-o**	***C. elegans*** **iPGM•Ce-2d**
**Data collection**			
Space group	$P2_1$	$P2_12_12_1$	$P2$
Cell dimensions			
a, *b*, *c* (Å)	67.85, 94.38, 102.2	70.32, 98.85, 73.10	73.78, 75.83, 101.42
α, β, γ (°)	90, 96.6, 90	90, 90, 90	90, 95.7, 90
Resolution (Å)	46.48-2.95 (3.13-2.95)	98.85-2.45 (2.54-2.45)	48.17-1.95 (1.99-1.95)
R_{sym} or R_{merge}	14.1 (62.5)	20.5 (84.0)	14.6 (60.3)
$I / \sigma I$	9.0 (2.3)	7.6 (1.9)	7.8 (2.2)
Completeness (%)	99.7 (99.9)	97.5 (99.4)	99.0 (98.1)
Redundancy	3.4 (3.5)	5.0 (4.7)	3.4 (3.4)
**Refinement**			
Resolution (Å)	42.98-2.95	40.44-2.45	35.49-1.95
No. reflections	26,992	43,673	80,411
R_{work} / R_{free} (%)	18.4/23.7	18.5/26.0	15.4/20.4
No. atoms			
Protein	7,837	7,886	8,001
Ligand/ion	0/4	0/4	210/4
Water	0	234	735
B-factors			
Protein	48.7	25.2	18.9
Ligand/ion	-/48.8	-/32.7	18.0/15.0
Water	-	20.9	26.3
R.m.s. deviations			
Bond lengths (Å)	0.008	0.009	0.017
Bond angles (°)	1.013	0.936	0.897

One single crystal was used for each data set. *Highest-resolution shell is shown in parentheses.

Supplementary Table 6. H-bonding and metal coordination distances in the *C. elegans* iPGM • **Ce-2d** structure

Ce-2d Residue or metal ion	iPGM Residue	Distance Min Residue Pair	Closest Atom Filter < 5Å	Ce-2d Residue or metal ion	iPGM Residue	Distance Min Residue Pair	Closest Atom Filter < 5Å
D-TYR 1	ASN336	3.80	O D-TYR1 /O ASN336	TYR 7	ARG284	2.42	CD1 TYR7/NH2 ARG284
D-TYR 1	PHE365	3.19	O D-TYR1 /CZ PHE365	TYR 7	ASP286	2.58	CE2 TYR7 /OD2 ASP286
D-TYR 1	GLY369	3.27	O D-TYR1 /O GLY369	TYR 9	VAL88	2.10	O TYR9/CG2 VAL88
D-TYR 1	GLY370	2.43	O D-TYR1 /O GLY370	TYR 9	ILE99	2.96	CG TYR9/CB ILE99
D-TYR 1	PHE366	2.74	CE1 D-TYR1 /CE1 PHE366	TYR 9	LEU 91	2.04	CE1 TYR9/CD2 LEU91
D-TYR 1	GLU 87	2.41	OH D-TYR1 /CB GLU87	TYR 9	ASN336	2.92	OH TYR9 /ND2 ASN336
D-TYR 1	LEU 91	3.76	OH D-TYR1 /CA LEU91	TYR 9	GLY369	3.61	OH TYR9 /C GLY369
ASP 2	ARG289	2.66	OD2 ASP 2 / NH2 ARG289	TYR 9	GLY370	3.31	OH TYR9/CA GLY370
TYR 3	ALA334	2.42	CD1 TYR3/N ALA334	LEU 10	ILE 99	3.32	N LEU10 /O ILE99
TYR 3	ARG289	4.54	CD2 TYR3/NE ARG289	LEU 10	LEU 78	2.28	CA LEU10 /CD2 LEU78
TYR 3	PRO333	2.41	CD2 TYR3/CB PRO333	LEU 10	LEU 82	3.26	O LEU10 /CD2 LEU82
TYR 3	GLN320	3.30	CE2 TYR3/O GLN320	LEU 10	TYR100	3.72	CD1 LEU10 /CD1 TYR100
TYR 3	ASP102	2.60	OH TYR3 /OD2 ASP102	LEU 10	GLN101	2.55	CD1 LEU10/OE1 GLN101
TYR 3	TYR283	3.06	OH TYR3/O TYR283	LEU 10	ILE103	4.05	CD1 LEU10/CD1 ILE103
TYR 3	ARG284	3.25	OH TYR3/C ARG284	LEU 10	PRO 79	2.95	CD2 LEU10/CD PRO79
TYR 3	ALA285	2.64	OH TYR3 /N ALA285	TYR 11	VAL 88	2.67	C TYR11/CB VAL88
TYR 3	ASP286	3.19	OH TYR3/N ASP286	TYR 11	ASN 85	1.90	O TYR11/CG ASN85
TYR 3	THR319	3.15	OH TYR3/CB THR319	TYR 11	HIS485	4.58	O TYR11/CE1 HIS485
PRO 4	ALA334	2.37	O PRO4/CB ALA334	TYR 11	GLU 87	3.02	OXT TYR11 /OE1 GLU87
PRO 4	ASN336	2.67	CG PRO4/CB ASN336	Mn2+	Asp426	2.14	Asp426/OD2
GLY 5	ILE 99	2.42	N GLY5/CG2 ILE99	Mn2+	His430	2.21	His430/NE2
GLY 5	TYR100	2.58	O GLY5/O TYR 100	Mn2+	His485	2.13	His485/NE2
GLY 5	GLN101	3.24	O GLY5/CA GLN101	Mn2+	H20	2.09, 2.45, 2.39	H20
GLY 5	ASP102	2.86	O GLY5/N ASP102	Zn2+	Asp37	1.95	OD1 Asp37
TYR 7	GLN101	2.73	CA TYR7 /NE2 GLN101	Zn2+	Ser86	1.99	OG Ser86
TYR 7	ILE 99	3.50	O TYR7/C ILE99	Zn2+	His468	2	NE2 His468
				Zn2+	Asp467	1.91	OD2 Asp467

Supplementary Methods

iPGM and dPGM Assays Phosphoglycerate mutase activity was measured either as a continuous or endpoint output assay. The continuous assay is based on lactate dehydrogenase oxidation of NADH as monitored at an absorbance of 340 nm using pyruvate supplied through a series of coupling enzymes as previously described for *C. elegans* and *B. malayi* iPGMs[1,2], adapted here in 1536-well microtiter plate format for the PGM orthologs and isozymes used in this study. Initial rate conditions determined from the continuous (kinetic) assay was used to calibrate an end-point bioluminescent assay format for a PGM profiling panel to evaluate the inhibitors developed in this project.

1536-well format kinetic assay Briefly, the forward glycolytic PGM catalyzed conversion of 3-phosphoglycerate (3-PG) to 2-phosphoglycerate was measured indirectly by monitoring the consumption of NADH through a coupled enzyme reaction. Four μL of the respective PGM enzyme was dispensed into black clear-bottom 1536 well plates (Cat# 789092-F, Greiner Bio-One North America) in a pH 8.0 assay buffer with the BioRaptor FRD (Beckman Coulter), for a final concentration of 30 mM Tris-HCl, 5 mM $MgSO_4$, 20 mM KCl and 0.08% BSA. Two μl of 3-PG substrate was added to each enzyme solution in a coupled enzyme assay buffer using the BioRaptor FRD, for a final assay concentration of 3 mM ADP, 500 μM NADH, 0.3 units enolase, 0.3 units pyruvate kinase, and 0.3 units lactate dehydrogenase. To confirm the 3-PG apparent K_M for several PGMs, 4 μL of *B. malayi* iPGM, *C. elegans* iPGM or *H. sapiens* dPGM enzymes were dispensed as above at a final concentration of 1 nM in pH 8.0 assay buffer. Two μl of an 11-point titration series of 3-PG substrate ranging from 0.083–2.0 mM was added to each enzyme solution in the coupled enzyme assay buffer. A 60 min time course was read for each enzyme-substrate solution at an absorbance of 340 nm on a Tecan Infinite M1000 PRO (Tecan Group Ltd), as shown in **Supplementary Fig. 3a**. NADH consumption was plotted as absorbance (340 nm) vs. time (s) in GraphPad Prism (GraphPad Software, Inc) for each of the enzyme-substrate titrations and the slope of the linear phase for each substrate concentration was calculated for the respective enzymes (**Supplementary Fig. 3b**). The initial rate (v_i) for each enzyme-substrate reaction was determined and plotted against molar substrate concentration to generate Michaelis-Menten curves, while the reciprocal of the rate and molar substrate concentrations were re-plotted as Lineweaver-Burk graphs in GraphPad Prism and apparent K_M values were estimated for each respective enzyme.

1536-well format luminescence assay ATP generated from the pyruvate kinase (PK) catalyzed conversion of phosphoenolpyruvate (PEP) to pyruvate was utilized to configure a luminescence output for the PGM enzyme panel. Various concentrations of iPGM and dPGM enzymes listed in **Supplementary Table 1** were dispensed in a total volume of 4 μl of the above assay buffer into respective wells of 1536-well white/solid bottom plates (Cat # 789173-F, Greiner Bio-One North America) using the BioRaptor FRD. Two μl of 3-PG substrate solution prepared at the estimated K_M concentration was added to each enzyme solution as described above, for a final assay concentration of 0.4 mM 3-PG, 3 mM ADP, 0.3 units enolase, and 0.3 units PK. Enzyme-substrate solutions were incubated at room temperature for 5 min, 4 μl Kinase-Glo Plus reagent (Promega Corporation, Madison, WI) was added to each reaction with the BioRaptor FRD, plates were incubated at room temperature for 10 min protected from light, and a luciferase-based ATP detection read-out was measured by a ViewLux plate reader (PerkinElmer, Waltham, MA). To expand the PGM enzyme selectivity panel to additional PGMs luminescence measurements for *O. volvulus* iPGM, *D. immitis* iPGM, *E. coli* iPGM and *E. coli* dPGM enzymes were measured as described above for each enzyme titrated from 1-20 nM in the presence of 0.4 mmol/L substrate. The enzyme concentration for each PGM that generated relatively equivalent luminescence RLU across the panel was selected for macrocyclic peptide and peptide analog profiling.

The pyruvate kinase (PK) coupling enzyme was included as an additional specificity control in the enzyme panel. PK concentrations of 0.3 units (~930 nM) or 0.15 units (~460 nM) were dispensed in a total volume of 4 μl of the above assay buffer into respective wells of 1536-well white/solid bottom plates as previously described. Two μl of PEP substrate solution prepared at an equivalent concentration to 3-PG substrate were added to the PK enzyme solution as described above, for a final assay concentration of 0.4 mM PEP and 3 mM ADP. The protocol for this assay profile panel is given in **Supplementary Table 1**.

SPPS cyclic peptides were tested under initial assay conditions described in **Supplementary Table 1** designed to give robust and uniform signal to background across the 7 enzyme PGM panel. Concentration response curves (CRCs) were fit using a 4 (see Online methods) or 5 parameter Hill equation (below). Model selection was determined by an extra-sum-of-squares F test for each ortholog condition. The majority of cyclic peptides displayed hyperbolic responses; those with steep Hill slopes or requiring a 5 parameter fit[3] were revaluated at assay conditions employing lower iPGM concentrations.

5 parameter Hill equation:

$$Y = \frac{(S_{max} - S_0)}{[1 + 10^{(n(LogXb) - X)}]^S}$$

Where

$$LogXb = LogEC50 + \frac{1}{n} Log\left(2^{\left(\frac{1}{S}\right)} - 1\right)$$

Where S0 is the signal at zero concentration, Smax is the signal at infinite concentration, n is the Hill slope, $LogEC_{50}$ is the log of the concentration at half-maximal signal, X is the log of the concentration, and S is the asymmetry parameter.

C. elegans iPGM titration The 1536-well format luminescence assay (above) was adapted to evaluate enzyme concentration on IC_{50} of **Ce-2**. *C. elegans* iPGM 10 nM to 50 pM concentration range was tested across a 16-pt 1:3 dilution of **Ce-2** from 3.83 μM to 0.27 pM final concentration, while 1 μM to 50 nM enzyme concentration range was tested across a 16-pt 1:2 dilution of Ce-2 from 3.83 μM to 117 pM final concentration. *C. elegans* iPGM concentrations of 1 nM–50 pM were incubated with 0.4 mM 3PG substrate solution for 15 min at room temperature and read on the ViewLux plate reader with standard assay settings (1 sec exp, medium gain, slow speed, 2X binning). 100 nM–5 nM enzyme concentrations were incubated with 0.4 m 3PG substrate solution for 5 min at room temperature and read on the ViewLux with standard assay settings. Enzyme concentrations of 500 nM and 1 μM were incubated with substrate for 5 min as above, but ViewLux plate reader settings were reduced to eliminate overexposure (1 sec exp, medium gain, medium speed, 2X binning). For the assay, 23 nL of the peptide titration series from a 16-point compound dispense plate were simultaneously transferred to 1536-well assay plates (Cat# 789092-F, Greiner Bio-One North America) using a 1536-pin tool (Wako) for a final concentration range of 3.8 μM–0.27 pM.

Gradient elution moving boundary electrophoresis (GEMBE) GEMBE was used for the direct monitoring of the activity of the enzyme iPGMs via label-free measurement of the substrate and product, 2-PG and 3-PG. A custom built apparatus was used to perform gradient elution moving boundary electrophoresis[4]. The separation channel consisted of a 5 cm length of capillary (360 μm OD, 15 μm ID) joining a custom machined 200 μL sample reservoir and 2000 μL buffer reservoir. Pressure in the headspace of the buffer reservoir was controlled using a Mensor Series 600 automated pressure calibrator. Platinum electrodes were inserted into the reservoirs to apply voltage across the separation channel. The capillary passed through a capacitively-coupled contactless conductivity detector (Tracedec) with the detection spot located approximately 2 cm from the sample reservoir. The buffer reservoir was filled with electrophoresis buffer (30 mM Tris-HCl pH 8.0, 20 mM $MgCl_2$), and enzyme reactions were mixed and run directly in the sample reservoir. The magnesium concentration in the electrophoresis buffer was chosen to optimize the resolution between the 2-PG and 3-PG signals[5] in the GEMBE electopherogram (**Fig. 1c** and **Supplementary Fig. 4**).

The equilibrium ratio of 2-PG to 3-PG predicted from the standard free energy is approximately 1:7 at room temperature[6]. Consequently, with the GEMBE assay, the typical change in signal is approximately 11x larger for the reaction starting with 2-PG and converting to 3-PG than for the reaction starting with 3-PG and converting to 2-PG. For the cofactor independent enzymes (*B. malayi* iPGM, *C. elegans* iPGM, and *E. coli* iPGM), the mutase reaction was found to be reversible in the GEMBE assay. Therefore, reactions with those enzymes were run starting with pure 2-PG and monitoring the conversion of 2-PG to 3-PG to maximize signal. For the cofactor dependent enzyme, *H. sapiens* dPGM, the mutase reaction was found to be irreversible in the GEMBE assay, with much faster reaction rates found for the conversion of 3-PG to 2-PG. Reactions with that enzyme were therefore started with pure 3-PG and the conversion of 3-PG to 2-PG was monitored.

The analytical separation of the product and substrate was carried out as follows. The buffer reservoir pressure was maintained at 30 kPa between separations and during sample loading. Once a sample was loaded and the GEMBE separation initiated (as described below), the pressure was reduced to 20 kPa for 30 s with the high voltage off. The high voltage (+2 kV) was then turned on, and the pressure was further reduced to a starting pressure of between 750 Pa and 2500 Pa and held constant for approximately 14 s. Note that the results presented here were obtained over several months using different capillaries with nominally identical properties. Because of slight differences in the electroosmotic properties and inner diameter of the capillaries, the optimal starting pressure varied between sets of analyses. The pressure was then reduced at a rate of 12.5 Pa/s until both 2-PG and 3-PG had been detected (216 s to 240 s). The pressure was then increase to 20 kPa and held constant for 10 s. The high voltage was turned off, and the pressure was increased to 30 kPa for at least 30 s before the start of the next GEMBE separation. The GEMBE separation was repeated 5 or 6 times for each sample, to monitor the conversion of substrate to product over a period of approximately 25 minutes.

Stock solutions of 2-PG and 3-PG were prepared at concentrations of 4 mM in electrophoresis buffer (30 mM Tris-HCl pH 8.0, 20 mM $MgCl_2$). Enzyme dilution buffer was prepared with 30 mM Tris-HCl pH 8.0, 20 mM $MgCl_2$, 6.4 mg/mL BSA. Inhibitor solutions were prepared in DMSO by 2-fold serial dilution to cover a range of at least 100-fold in concentration. PGM enzyme stock solutions were in 50% glycerol and were stored at -20 ^oC. Final enzyme concentrations used were chosen to give similar reaction rates for the GEMBE assays. Before each enzyme reaction mixture was loaded, the sample reservoir was rinsed with electrophoresis buffer.

For the GEMBE measurements with **Ce-2** and **Ce-2d**, the enzyme reactions were mixed and initiated according to the following: working solutions of *C. elegans*, *B. malayi* and *E. coli* iPGM and *H. sapiens* dPGM were prepared by

volumetric dilution from 93, 136, 62 and 125 μM stocks with enzyme dilution buffer to enzyme concentration of 47, 34, 250 and 21 nM respectively. To initiate a reaction, 159 μL of electrophoresis buffer was added to the sample reservoir, followed by 1 μL of inhibitor solution in DMSO (or pure DMSO for no-inhibitor controls) and 20 μL of the enzyme working solution. Mixing was achieved with vigorous pipetting. Five minutes after addition of the enzyme, 20 μL of substrate (2-PG or 3-PG) stock solution was added, and the sample was again mixed with pipetting. Forty-five seconds after addition of the substrate, the first analytical separation was started. The final concentrations of all components in the reaction were: 30 mM Tris HCl, 20 mM $MgCl_2$, 0.64 mg/mL BSA, 400 μM substrate, 0.5 %v/v DMSO, inhibitor ranging from 195 pM to 2.5 μM, and either 3.4 nM *B. malayi* iPGM, 4.6 nM *C. elegans* iPGM, 25 nM *E. coli* iPGM, or 2.1 nM *H. sapiens* dPGM.

Analysis of the GEMBE data and calculation of reaction rates was similar to that previously reported[7]. Briefly, the detector signal *vs*. time data for each electropherogram (see **Supplementary Fig. 4a**, for example) was fit to a functional form consisting of the sum of 3 complementary error functions and a quadratic baseline:

$$signal(t) = A_0 + A_1 t + A_2 t^2 + \frac{C_1}{2}\mathrm{erfc}\left(\frac{t - t_1}{\sqrt{2}\sigma_1}\right) + \frac{C_2}{2}\mathrm{erfc}\left(\frac{t - t_2}{\sqrt{2}\sigma_2}\right) + \frac{C_3}{2}\mathrm{erfc}\left(\frac{t - t_3}{\sqrt{2}\sigma_3}\right).$$

The 3 error functions correspond to 2-PG, 3-PG, and an unknown species present in the enzyme stock solutions. Calibration measurements indicated that the resulting best fit values for C_2 and C_3 were proportional to the concentration of the analytes, 2-PG and 3-PG, respectively. The percent conversion was then calculated from:

$$percent\ conversion = \frac{C_3}{C_2 + C_3} \cdot 100.$$

The reaction rate was determined by the slope of a linear fit to the percent conversion *vs*. reaction time data for the first 4 GEMBE separations with each sample. The reaction rate was normalized by the rate of reaction from a no inhibitor control. Data was modeled to a four (see Online methods) or five parameter Hill equation for initial estimation of IC_{50}s using Prism GraphPad (*see above*).

Size exclusion chromatography Samples were analyzed and fractionated on a Superdex 75 16/600 column using an AKTA Pure system. Samples, 500 μL, were eluted at 1 mL/min in buffer containing 30mM Tris, 150 mM NaCl and 2 mM $MgSO_4$ at 4^0C. Elution profile absorbance recorded with in-line detection at 280 nm, and 1 or 2 mL fractions were collected in 96 deep-well plates.

Polyacrylamide gel electrophoresis Protein (10μL/lane) was electrophoresed on Criterion TGX 4-12% precast polyacrylamide mini slab gels (Bio-Rad) in 1x Tris running buffer, ambient temperature at 200V for 45 min. Samples, 100 μL aliquots from size exclusion column fractions plus 33 μL 4x Laemmeli sample buffer (Bio-Rad 161-0747) ±2% βME were heated for 10 min at 95^0C. Gels were fixed for 30 mins in 50% methanol 10% acetic acid then stained with Coomassie Brilliant Blue G-250 colloidal protein stain (Sigma-Aldrich B8522) overnight and imaged on a Bio-Rad ChemiDoc imager. Molecular weight ladder (Bio-Rad #161-0376).

Solid phase peptide chemical synthesis (SPPS) All peptides were chemically synthesized on a 25 μmole scale using a Syro Wave automated peptide synthesizer (Biotage) by Fmoc SPPS. Firstly, NovaPEG Rink Amide resins were incubated

with N,N-dimethylformamide (DMF) with rotation at ambient temperature for 30 min and washed 5 times with DMF. Coupling of each Fmoc-protected amino acid was performed on the engorged resin with a solution of 300 μL 0.5 M Fmoc-protected amino acid, 300 μL 0.5 M 2-(1H-Benzotriazole-1-yl)-1,1,3,3-tetramethyluronium hexafluorophosphate (HBTU) and 1-hydroxybenzotriazole (HOBt), and 150 μL 0.5 M N,N-diisopropylethylamine (DIPEA) in DMF and reacted for 1 hour at ambient temperature. After washing the resins with 1 mL DMF five times, Fmoc-deprotection was performed by incubating the resin with 600 μL 40% piperidine in DMF (vol/vol) and reacted for 30 min at ambient temperature. Each peptide was synthesized using the appropriately protected amino acid monomers corresponding to sequences in **Tables 1** and **2** by repeating the Fmoc-protected amino acid coupling and Fmoc-deprotection steps accordingly. The N-terminal α-amino group of the synthesized peptides on the resin was chloroacetylated by incubating with a solution of 500 μL 0.5 M chloroacetyl N-hydroxysuccinimide (NHS) ester in N-methylpyrrolidone (NMP) with rotation for 60 min at ambient temperature. For the synthesis of **Ce-L2** and **Ce-L2d**, the N-terminal α-amino group was acetylated by incubating with a solution of 500 μL 0.5 M acetic anhydride and 0.25 M DIPEA in NMP with rotation for 60 min at ambient temperature. After washing the resin with 5 x 1 mL DMF, peptides were fully deprotected and cleaved from resin by incubating with a solution of 2 mL trifluoroacetic acid (TFA), water, triisopropylsilane (TIS) and ethanedithiol (EDT) (92.5:2.5:2.5:2.5) with rotation for 3 hours at ambient temperature and precipitated with diethyl ether. The peptide pellet was dissolved in 10 mL DMSO/0.1%TFA in water (1:1), and the pH adjusted to >8 by addition of triethylamine (TEA), and incubate at ambient temperature for 1 h to enhance the cyclization via a thioether bond formation between N-terminal chloroacetamide group and cysteine sulfhydryl group. Peptide mass and cyclization was confirmed by MALDI-TOF MS analysis. The cyclization reaction was quenched by addition of TFA to acidify the peptide suspensions. Peptides were then purified by reverse-phase HPLC (**Supplementary Table 4** and **Fig. 17,** *left panel*), molecular masses were verified by MALDI-TOF MS analysis (**Supplementary Table 4** and **Fig. 17,** *right panel*), using a microflex or autoflex instrument (Bruker Daltonics). Ring junction confirmed by MSMS spectrum and fragment analysis (**Supplementary Fig. 18**)

Crystallographic Analysis *Ce iPGM modeling:* The subunits of iPGM-o were structurally very similar with an RMSD deviation of 0.77 Å between Cα atoms for 520 residues aligned. In addition, the structures of iPGM-o and iPGM-m were quite similar with an RMSD deviation of 1.85 Å between Cα atoms for 495 residues aligned. However, one can clearly see that the phosphatase domains are much more closely aligned whereas the transferase domains exhibit a slight shift (**Supplementary Figure 9c**) which is likely due to the conformational flexibility of this domain. The structure of iPGM-o also contains two metal ions in the substrate binding site which bind in a similar manner to iPGM-m (see below).

Comparison of C. elegans iPGM-m to other apo iPGM structures Superposition of *C. elegans* iPGM-m with iPGM from *Bacillus anthracis* (PDB: 2IFY) yielded an RMSD of 2.76 Å between Cα atoms (476 residues aligned). Although the deviation from superposition is somewhat large between the two structures, the overall fold is very similar between the two structures (**Supplementary Figure 11**). The structure of *C. elegans* iPGM-m was also compared with that of a substrate bound iPGM from *Bacillus stearothermophilus* (PDB: 1O98). Superposition yielded an RMSD of 1.07 Å between Cα atoms. However, only 282 residues could be aligned for residues in the transferase domain since substrate binding produces a large conformational change in the phosphatase domain (**Supplementary Figure 11b**).

Metal ion sites: Large peaks of positive electron density (Fo-Fc) were observed in the metal binding sites of the phosphatase domain following refinement **Supplementary Figure 12a**. This region is occupied by Asp 426 and His 430

(site1) and Asp 37, Ser 86, Asp 467 and His 468 (site2). When these sites were refined as Mg^{2+} ions, residual positive electron density was observed suggesting that a larger metal ion is present. Modelling Mn^{2+} ions at each site resulted in a *B*-factor of approximately 17 $Å^2$ at site1, but site2 contained some positive electron density and a *B*-factor of approximately 7 $Å^2$. Additionally, the coordinating distances between the surrounding residues and the ion at site 2 were approximately 1.9 Å to 2.0 Å which are shorter than expected for a Mn^{2+} ion (2.1 Å to 2.2 Å). Phased anomalous difference maps were calculated, using data collected at a wavelength of 1.0000 Å, which yielded peak heights of: site 1 (5.7σ) and site 2 (11.7σ) as shown in **Supplementary Figure 12a**. Zn^{2+} ions may occupy site 2 based on the coordinating distances and anomalous signal, and homology of the iPGM phosphatase domain to that of *E. coli* alkaline phosphatase[8]. The theoretical anomalous signal at a wavelength of 1.0000 Å, is 2.6 e^- and 1.4 e^- for Zn^{2+} and Mn^{2+} ions respectively. Additionally, data were collected a wavelength of 1.9016 Å which is on the low energy side of the Mn^{2+} absorption edge. The theoretical anomalous signal at this wavelength is 1.1 e^- and 0.49 e^- for Zn^{2+} and Mn^{2+} ions respectively. A phased anomalous difference map was calculated using the 1.9016 Å data and produced no peaks at site 1 whereas peaks of approximately 5σ were observed at site 2. Subsequent refinement of Mn^{2+} and Zn^{2+} ions at site1 and site2 respectively yielded no residual positive electron density which further supported our assignments for these sites. The coordination of these metals by *C. elegans* iPGM is shown in **Supplementary Figure 12b** and the distances are listed in **Supplementary Table 6**. It should be noted that an Mg^{2+} binding site, from the crystallization solution, was also observed as shown in **Supplementary Figure 12c**.

***C. elegans* viability assay** *C. elegans* strains N2 (wild type) was used for compound testing. Worms were handled using standard methods. They were cultivated on nematode growth medium (NGM) plates in a 20°C incubator and fed on living *Escherichia coli* strain OP50. The *E. coli* OP50 cells were precultured overnight at 37°C before spreading on the surface of NGM plates. Synchronous liquid culture of L1 *C. elegans* obtained through egg bleaching and individual L4 picked from NGM plates under the microscope were used for testing. Either 20 L1s or 1 L4 were placed in individual well of 96-well plate in 100uL of S medium supplied with dead *E. coli* HB101 as food source. Compounds were added into the well at different concentrations and incubated with worms at 20°C for up to 7 days. The effect of compounds on worm health was measured by food consumption, development and reproduction. Food consumption was measured by a decline in OD_{600} nm readings monitored using a SpectraMax M5 microplate reader. Worm development and F1 progeny production were monitored visually under a microscope.

Supplementary methods references

1. White, M.F. & Fothergill-Gilmore, L.A. Development of a mutagenesis, expression and purification system for yeast phosphoglycerate mutase. Investigation of the role of active-site His181. *Eur J Biochem* **207**, 709-714 (1992).
2. Zhang, Y., Foster, J.M., Kumar, S., Fougere, M. & Carlow, C.K. Cofactor-independent phosphoglycerate mutase has an essential role in Caenorhabditis elegans and is conserved in parasitic nematodes. *J Biol Chem* **279**, 37185-37190 (2004).
3. Giraldo, J., Vivas, N.M., Vila, E. & Badia, A. Assessing the (a)symmetry of concentration-effect curves: empirical versus mechanistic models. *Pharmacol Ther* **95**, 21-45 (2002).
4. Strychalski, E.A., Henry, A.C. & Ross, D. Expanding the capabilities of microfluidic gradient elution moving boundary electrophoresis for complex samples. *Analytical chemistry* **83**, 6316-6322 (2011).

5. Schaeper, J.P., Shamsi, S.A. & Danielson, N.D. Separation of phosphorylated sugars using capillary electrophoresis with indirect photometric detection. *J Capillary Electrophor* **3**, 215-221 (1996).
6. Clarke, J.B., Birch, M. & Britton, H.G. The equilibrium constant of the phosphoglyceromutase reaction. *Biochem J* **139**, 491-497 (1974).
7. Ross, D. & Kralj, J.G. Simple device for multiplexed electrophoretic separations using gradient elution moving boundary electrophoresis with channel current detection. *Anal Chem* **80**, 9467-9474 (2008).
8. Jedrzejas, M.J. Structure, function, and evolution of phosphoglycerate mutases: comparison with fructose-2,6-bisphosphatase, acid phosphatase, and alkaline phosphatase. *Progress in biophysics and molecular biology* **73**, 263-287 (2000).
